# Key characteristics of palliative care integration in intensive care units (ICUs): A scoping review

**DOI:** 10.1016/j.ijnsa.2026.100535

**Published:** 2026-04-08

**Authors:** Yvonne King, Peter May, Barbara Whyte, Eóin Tiernan, Anne-Marie Brady

**Affiliations:** a Trinity Centre for Practice & Healthcare Innovation, School of Nursing and Midwifery, Trinity College Dublin, 24 D’Olier Street, Dublin 2, Ireland; b Cicely Saunders Institute of Palliative Care, Policy & Rehabilitation, Florence Nightingale Faculty of Nursing, Midwifery & Palliative Care, Kings College London; c St Vincent’s University Hospital, Elm Park, Dublin 4, Ireland

**Keywords:** Critical Care, End-of-Life Care, Intensive Care Units, Palliative Care, Scoping Reviews

## Abstract

**Background:**

Intensive care units (ICUs) are increasingly managing patients with greater clinical complexity and life-limiting conditions. Systematic review evidence highlights that early palliative care, integrated alongside active treatment and not solely at end-of-life, can significantly improve outcomes for both patients and their families. Despite evidence on the effectiveness of palliative care integration models in the ICU setting, the specific characteristics for integration remain poorly articulated and insufficiently understood.

**Objectives:**

To systematically map key characteristics of palliative care integration models in adult ICUs.

**Eligibility criteria:**

We included integration models/frameworks for palliative care in adult ICUs and excluded studies that reported on stand-alone interventions. We limited the search to studies published from 2015, without restriction on study design or geographic location, to ensure a broad and inclusive review of global evidence.

**Sources of evidence:**

A comprehensive search was performed in July 2024 across six data bases: PsycINFO, CINAHL, Excerpta EMBASE, Cochrane Library, MEDLINE, and Web of Science in July 2024**.** The keywords ‘palliative care’ and ‘intensive care unit’ were used to identify literature on palliative care integration in ICU.

**Charting Methods:**

A data extraction table was used to extract relevant information from the included original research and review articles. Data were analysed using thematic analysis approach.

**Results:**

We screened 10,262 titles/abstracts, read 980 full-text articles, and ultimately included 60 publications in the final analysis. Thirty-five were original research studies and 25 were review articles, including four systematic reviews. Most research studies originated from the United States (n = 32), with one study each from India, Canada, and Singapore. We identified four key characteristics essential for integrating palliative care in the ICU including:

1. Screening criteria: structured tools to identify patients who may benefit from early palliative care alongside ongoing treatment.

2. Embedded daily practices: routines focused on symptom assessment, timely family meetings, and proactive palliative needs evaluation.

3. Education and training: targeted programs to strengthen ICU clinicians’ understanding of palliative care beyond end-of-life.

4. Specialist palliative care collaboration**:** joint rounding, embedded palliative nurse practitioners, and coordinated teamwork between ICU and specialist palliative care teams.

Facilitators included embedding education in daily ICU practice and system-wide alignment, while barriers included limited clinician knowledge and the perception of palliative care as end-of-life only.

**Conclusion:**

This scoping review provides a comprehensive overview of key characteristics of models integrating palliative care into adult ICUs, informing development, implementation, standardisation, and policy decisions.

**Registration:**

Open Science Framework: DOI 10.17605/OSF.IO/CZS26.

## Highlights

• Palliative care in intensive care units (ICUs) can reduce symptom burden, improve patient-centred care, support families during critical illness and provide continuity of care after ICU discharge.
• Early palliative care alongside life-prolonging treatment is recommended for patients with serious illnesses, and effective integration models are available.
• However, key characteristics of palliative care integration models in ICUs remain poorly defined.
• This review identifies four key characteristics of palliative care integration models in adult ICUs: screening criteria, embedded palliative care practices, education, and specialist palliative care collaboration.
• The findings highlight how structured approaches to integrating palliative care support earlier identification of needs and improve care coordination in ICUs.
• The review also identifies gaps in international evidence, with most studies originating from the United States and limited research examining integration models in other healthcare systems.

**What is already known**

• Palliative care in intensive care units (ICUs) can reduce symptom burden, improve patient-centred care, support families during critical illness and provide continuity of care after ICU discharge.
• Early palliative care alongside life-prolonging treatment is recommended for patients with serious illnesses, and effective integration models are available.
• However, key characteristics of palliative care integration models in ICUs remain poorly defined.

Alt-text: Unlabelled box dummy alt text

**What this paper adds**

• This review identifies four key characteristics of palliative care integration models in adult ICUs: screening criteria, embedded palliative care practices, education, and specialist palliative care collaboration.
• The findings highlight how structured approaches to integrating palliative care support earlier identification of needs and improve care coordination in ICUs.
• The review also identifies gaps in international evidence, with most studies originating from the United States and limited research examining integration models in other healthcare systems.

Alt-text: Unlabelled box dummy alt text

## 1 Background

Every year, millions of people are admitted to intensive care units (ICU) worldwide, with a reported mortality rate of 20–30% (Adhikari et al., 2010; Vincent et al., 2014). Most patients are over 60 and face a higher risk of death due to age-related decline, multimorbidity, and frailty (Athari et al., 2018; Akinosoglou et al., 2023; Gonçalves-Pereira et al., 2023). As a result, they often experience prolonged hospital stays and have complex needs. This highlights the importance of initiating early goals-of-care discussions to support effective communication, ensuring that decisions reflect patients’ values and minimise non-beneficial treatments (Araujo, et al., 2023; Ma et al., 2019)

For those who survive, recovery can be prolonged and accompanied by psychological and functional impairments (Geense et al., 2021; Rai et al., 2020). Evidence shows that patients in ICU frequently experience unmet needs and symptom burden, including refractory pain, shortness of breath, and thirst (Puntillo et al., 2014; Su et al., 2018). Communication challenges, conflicts in decision-making, and the use of non-beneficial treatments further exacerbate these unmet needs. These challenges predated the COVID-19 pandemic. However, this crisis significantly amplified them, as ICUs faced unprecedented admissions rates and increased strain on staff, patients, and their families (Nugent et al., 2023; Tan et al., 2021).

### 1.1 Palliative care in intensive care units

Palliative care in ICU offers a structured response to these challenges. Integrating palliative care early can reduce refractory symptoms, enhance early goals-of-care discussions, align care with values, and support complex decision-making (Aslakson et al., 2014; Grouls et al. 2022; Michels et al., 2023). While specialists’ involvement ensures continuity of care from ICU to the community (Aslakson et al., 2014). In this way palliative care aims to prevent and relieve suffering through early identification, assessment, and treatment of physical, psychosocial, and spiritual needs, providing essential support to patients and their families (Curtis et al., 2022).

Although palliative care and end-of-life-care aim to improve quality of life (World Health Organisation [WHO], 2020), they differ in timing and treatment options. End-of-life-care aims to enhance dignity and comfort in the final stages of life and is a subset of palliative care (Curtis et al., 2022; Wiencek, 2024). Whereas palliative care can be integrated alongside curative treatments throughout Illness trajectories regardless of prognosis, including during ICU care (European Association of Palliative Care [EAPC, 2019]; WHO, 2020).

### 1.2 Challenges to Integration

Despite this, palliative care in ICUs is often limited to end-of-life care (Helgeson et al., 2023; Kodadek et al., 2023). Misconceptions that end-of-life care accelerates death remain a barrier (Hardin & Gonzales, 2015; Soper et al., 2023). Yet, randomised controlled trials (RCT), demonstrate that early integration does not hasten death (White et al., 2018; Michels et al., 2023). Instead, it improves patient-centred care, strengthens clinician-family communication, supports shared decision-making, and is associated with shorter ICU stays, which reduces costs. While continuing palliative care after ICU discharge may reduce inappropriate hospital visits (Eaton et al., 2023; Bevins et al., 2021). Reflecting this evidence, WHO (2020) and critical care societies such as the American Association of Critical-Care Nurses (AACN) advocate for early palliative care alongside treatment for people with serious illness, including those in the ICU (Baker et al., 2015).

### 1.3 Post COVID changes and Opportunities

During the COVID-19 pandemic, clinicians increasingly relied on advanced ICU interventions, such as ventilatory support and extracorporeal membrane oxygenation (Czyż-Szypenbejl et al., 2022). Although these therapies prolonged life for many critically ill patients, they also introduced greater complexity into clinical decision-making and necessitated more rigorous evaluation of interventions that might offer limited or no benefit (Czyż-Szypenbejl et al., 2022). This pandemic further highlighted the importance of early goals-of-care discussions which have been shown to reduce non-beneficial interventions, and improve quality of life (Akinosoglou et al., 2023; Brunker et al., 2023). Such discussions are especially critical in the ICU, where the acute and unpredictable nature of illness trajectories requires timely and proactive communication (Schoenherr et al., 2020). Evidence also indicates that when goals-of-care are not adequately addressed, clinicians are more likely to experience emotional exhaustion and compassion fatigue (Roczen et al., 2016; Wolf et al. 2016). Collectively these factors highlight the necessity of prioritising early palliative care integration within ICU settings.

### 1.4 Rationale for Review

The rapid expansion of ICU-based palliative care programs in the United States has led to the development of various integration models to guide implementation (Adler et al., 2017; Metaxa et al., 2021; Takahashi et al., 2024). Despite these advancements, the specific characteristics of these models remain poorly understood and inconsistently reported. This lack of clarity poses significant challenges to the successful translation and impact of palliative care in ICU settings, reinforcing its importance as a research priority (Aslakson et al., 2017). Given the heterogeneity and exploratory nature of this issue, we conducted a scoping review to systematically map the key characteristics of existing integration models**.**

## 2 Methods

This scoping review followed Arksey and O’Malley (2005) methodological framework with additional guidance from the Joanna Briggs Institute methodology for scoping reviews (Peters et al., 2020). DOI 10.17605/OSF.IO/CZS26.

### 2.1 Search Strategy

**Figure 1:**
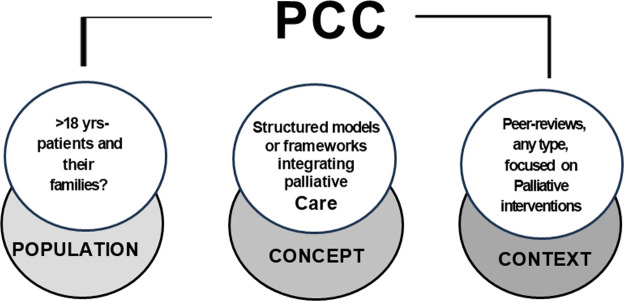
Population, concept & context.

We used the Population, Concept, and Context (PCC) mnemonic, see Figure 1. Search terms were developed using this PCC framework in collaboration with an information retrieval specialist (JEC), which included “intensive care units,” and “palliative care,” as well as related MeSH terms, truncations, and synonyms (see Supplement File S1). YK systematically searched CINAHL, MEDLINE, EMBASE, PubMed, PsycINFO, and the Cochrane Library, using Boolean operators (‘OR’ for synonyms/index terms and ‘AND’ to combine key terms). We also reviewed grey literature sources, including the Grey Literature Report, Google Scholar, and Open Grey. Additionally, YK manually searched relevant journals and websites, including the United States ’s Center to Advance Palliative Care (CAPC). We undertook the search between July 1 and July 24, 2024 (CAPC, 2019).

### 2.2 Study Selection

Improving palliative care in acute settings requires a whole-systems approach. This review focused on models or frameworks for integrating palliative care within adult ICUs, deliberately excluding studies that reported on stand-alone interventions. See Table 1 for the inclusion/exclusion criteria. To maintain conceptual clarity, only references that explicitly distinguished palliative care from end-of-life care were included. This ensured consistent data on the characteristics of palliative care systems integrated alongside curative treatment in the ICU, aligning with this review’s objectives. While end-of-life care remains an essential component of palliative care, our emphasis was on its earlier introduction to patients living with a life-limiting illness and who continue to receive curative treatment. This focus enabled a targeted examination of systematic approaches necessary for sustainable, coordinated care delivery. We included all peer-reviewed publications (reviews and empirical research). To ensure contemporary evidence all studies published prior to 2015 were excluded. This approach encompassed pre-COVID 19 studies, which was essential given many interventions reported in post-pandemic publications were developed or implemented before COVID-19 or were applied to a broader ICU population. Including pre-pandemic studies allowed the capture of foundational evidence and integration strategies that remain pertinent to current ICU palliative care practices. To ensure a comprehensive perspective consistent with scoping review methodology we did not apply geographic or language limitations. However, we restricted our inclusion to studies conducted in adult intensive care units, excluding those focused on intensive palliative care units or paediatric/neonatal ICUs.

**Table 1:** Inclusion & Exclusion criteria.

Category	Inclusion Criteria	Exclusion Criteria
**Studies**	Peer-reviewed publications using any research method evaluating or describing structured models integrating palliative care into intensive care. We also included peer review literature reviews, and systematic reviews.	Publications where "palliative care" is not explicitly mentioned, or when only end-of-life care initiatives were discussed, or non-peer-reviewed publications. Published before 2015.
**Settings**	Adult intensive care units, including general, cardiac, surgical, medical, neurological, and burns intensive care units.	Paediatric/neonatal intensive care units and intensive palliative care units.
**Interventions**	Structured models or frameworks integrating palliative care into intensive care units as an overall approach.	Individual palliative care initiatives not part of a comprehensive model.
**Population**	Patients aged ≥18 years in adult intensive care units and their families.	Patients aged <18 years.

### 2.3 Quality appraisal

Scoping reviews aim to map the breadth and characteristics of available evidence rather than evaluate intervention effectiveness or methodological quality. Guidance from the Joanna Briggs Institute and others indicates that critical appraisal is optional and should be guided by the review objectives (Arksey & O’Malley, 2005; Peters et al., 2020). As this review sought to map key characteristic of structured models for integrating palliative care within ICU, studies were included irrespective of methodological design or quality to ensure comprehensive coverage of the available literature.

### 2.4 Data Extraction

We mapped the key characteristics of palliative care integration across different types of peer-reviewed studies. Two reviewers (YK & BW) independently screened all publications using a screening protocol according to the eligibility criteria. We resolved differences of opinions through discussion, with a third person (AMB) when needed. A data extraction table was used to extract relevant information from both original research and review articles. From the 35 research studies, we recorded author names, publication year, country, ICU type, study design, objectives, duration, intervention type, sample size, and intervention characteristics. For the 25 review articles, we collected author details, publication year, review aims, and significant discussions related to palliative care integration models and frameworks. This approach enables the comparison of key features and methods across studies. providing a comprehensive picture of key characteristics of models used to integrated palliative care into ICU settings.

### 2.5 Thematic Analysis

Given the heterogeneity of included studies, a meta-analysis was not feasible. We therefore used Braun and Clarke’s (2021) thematic analysis approach. Initial inductive coding of the 35 research studies allowed themes to emerge inductively from the data. These preliminary themes then guided both inductive and deductive coding across all 60 studies, enabling refinement while remaining open to new insights. The process involved repeated reading, extraction of key phrases, and development of initial codes which are subsequently clustered into broader categories. These were iteratively reviewed, refined and named to capture core characteristics analysis of models integrating palliative care integration models. The research team met regularly to discuss and agree on codes and themes, ensuring consensus and consistency in interpretation. Table 3 presents a representative thematic

## 3 Results

### 3.1 Search Results

**Figure 2:**
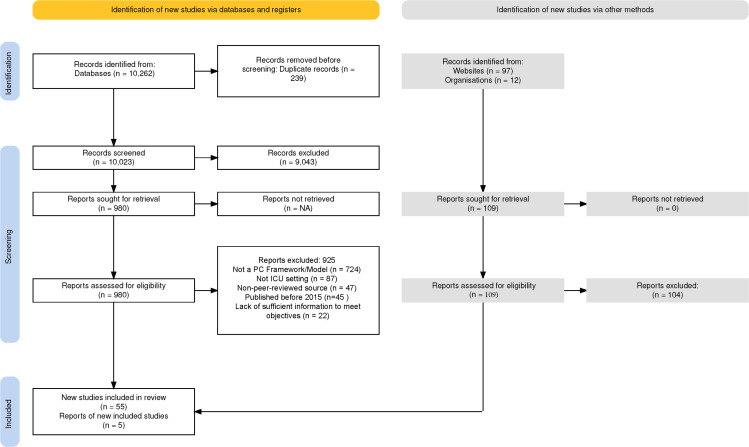
PRISMA Flow Chart.

We identified 10,262 publications and exported them into Covidence. After removing 239 duplicates, YK and BW independently screened 10,023 titles and abstracts and reviewed 980 full text publications. Fifty-six met the inclusion criteria. Reasons for exclusion included wrong setting, publication before 2015, non-review studies, abstracts, and studies describing single palliative care initiatives (e.g., bereavement or communication strategies) rather than integrated care models. Further details for the reasons of exclusion are found in the PRISMA (see Figure 2). We found five from a manual search. We included 35 research studies (2015-2023), and 25 review studies (2015-2024), totalling 60. Although no language restrictions were applied during the search process, all included studies were available in English.

### 3.2 Study characteristics

**Figure 3:**
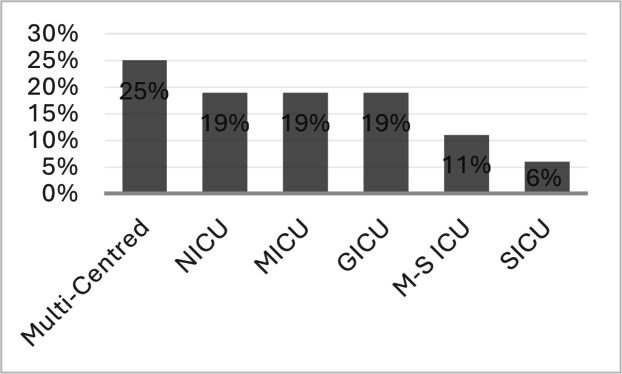
Distribution of ICU Types. **Note.** GICU = General Intensive Care Unit; MICU = Medical Intensive Care Unit; NICU = Neurological Intensive Care Unit; SICU = Surgical Intensive Care Unit; M-S ICU = Medical-Surgical Intensive Care Unit. Multi-centred refers to studies across more than one ICU type or hospital site.

**Figure 4:**
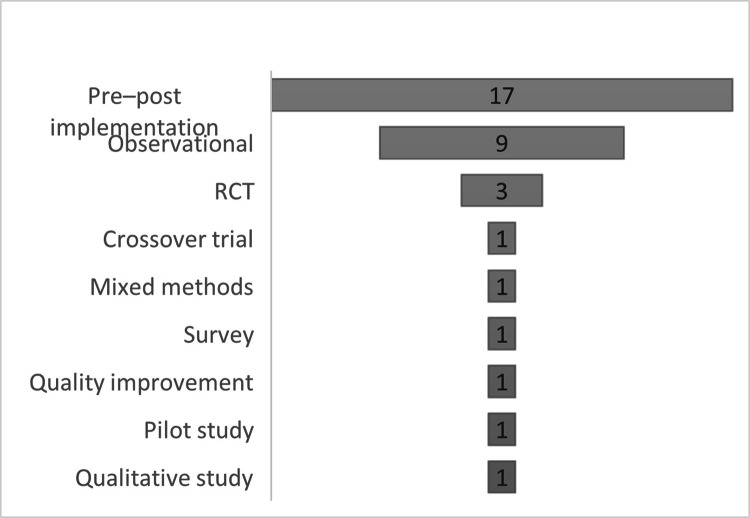
Research Study Designs (N=35). * Observational= prospective, retrospective, chart review, case–control, Randomised controlled trial=RCT.

We included 35 research studies and 25 review studies (see Table 2). Research studies originated predominantly from the United States (US) of America, accounting for 32 of the included studies, with one study each from India (Rao et al., 2023), Canada (Kyeremanteng et al., 2020), and Singapore (Poi et al., 2022). These studies represented a broad range of ICU settings. Multi-centre ICUs were the most common (25%), followed by general, medical, surgical, and neurological ICUs, each representing approximately 19% of the sample (Figure 3). Six studies specifically focused on the COVID-19 populations (Davila et al., 2023; Grouls et al., 2022; Liu et al., 2022; Rao et al., 2023; Schockett et al., 2021; Schoenherr et al., 2020). Regarding methodology, many included research studies (n=17) employed pre–post-intervention approaches, followed by observational methods (n=10) (see Figure 4). Only three studies used RCTsand one employed cross-over trial design (Carson et al., 2016; Cox et al., 2018; Helgeson et al., 2023; Ma et al., 2019). This predominance of pre–post and observational methods, with relatively few RCTs, reflects the early stage of development and implementation of ICU palliative care integration research.

**Table 2:** Research studies & Review studies.

Research Studies									
Ref	References		Country	Design	ICU Type	Aim	Months	Model & Sample	Intervention Characteristics
1	Creutzfeldt et al., 2015		USA	Prospective cohort	NICU	Measure prevalence of needs	6	Integrative, N = 262	Embedded PC practices: PC Needs Screening Tool (PNST) & action items to address identified needs.
2	Jenko et al., 2015		USA	Pre-Post	MICU	Measure impact	6	Integrative, N = 26	Clinician education, screening criteria, nurse engagement activities.
3	Carson et al., 2016		USA	RCT	Multi-Centred	Measure impact	25	Consultative, N = 256	Time structured family meetings led by SPC, family pamphlets, SPC support/consultations.
4	Constantine et al., 2016		USA	Pre-Post	Multi-Centred	Measure impact	6	Integrative, N = 55	Time embedded PC practices, clinician education, C&CB.
5	Braus et al., 2016		USA	Pre-Post	GICU	Measure impact	12	Consultative, N = 203	Joint rounding, screening criteria, SPC support/consultation.
6	Mun et al., 2016		USA	Pre-Post	GICU	Measure impact	6	Integrative, N = 392	Screening criteria, time embedded PC practices, SPC support/consultation, patients/families’ videos.
7	Anderson et al., 2017		USA	Pre-Post	Multi-Centred	Measure impact	30	Integrative, N = 428	Clinician education, ANP/nurse leaders, embedded PC practices e.g., daily PC assessments.
8	Henderson et al., 2017		USA	Observational prospective	MICU	Analyse use of the Rotherman Index and LOS	3	Consultative, N = 96	Screening criteria, SPC support/consultation.
9	O'Mahony et al., 2017		USA	Case-Control	Multi-Centred	Measure impact	12	Consultative, N = 62	ANPs, joint rounding, clinical education, ANP & ICU consultant communication, screening criteria, SPC support/ consultations.
10	Zalenski et al., 2017		USA	Pre-Post	Multi-Centred	Measure impact	4	Consultative, N = 649	Screening criteria, SPC & ICU consultant communication.
11	Cox et al., 2018		USA	Pre-Post	GICU	Develop PC app & measure impact	24	Integrative, N = 116	App platform- screening-criteria, family reporting system for PC needs, timed PC needs review & discussion. family meetings led by SPC, nurse champion, SPC support/consultation.
12	McCarroll, 2018		USA	Pre-Post	M-S ICU	Measure impact	6	Consultative, N = 20	Screening criteria, nurse led, clinician education, champions, SPC support.
13	Mun et al., 2018		USA	Pre-Post	M-S ICU	Improve ICU PC	3	Combined, N = 392	Screening criteria, C&CB bundle.
14	Ma et al., 2019		USA	Randomised crossover	Multi-Centred	Measure impact	10	Consultative, N = 199	Screening criteria, SPC consultation within 48 hrs.
15	Vig 2019		USA	Pre-Post	MICU	Measure impact	24	Consultative, N = 400	Weekly PC & ANP undertaking ethics rounding with ICU team, clinical education, debriefing sessions.
16	Vuong et al., 2019		USA	Pre-Post	MICU	Measure impact	24	Integrative, N = 3424	C&CB incl. time embedded PC practices, family pamphlets, champions.
17	Kyeremanteng et al., 2020		Canada	Survey	Multi-Centred	Identify barriers of ICU PC	5	Consultative, N = 181	Screening criteria, SPC support/consultation, role modelling EOLC communication by SPC, checklist of EOLC discussion points, clinical education on PC principles, SPC joint rounding, Need for emotional ICU clinician support.
18	Martz et al., 2020		USA	Retrospective	NICU	Measure impact	24	Integrative, N = 113	C&CB embedded practice adapted to include a SPC referral by day 5, informational leaflets, clinician education.
19	Schoenherr et al., 2020		USA	Pilot	GICU	Identify PC needs	1.5	Consultative, N = 29	Screening criteria, SPC reviewed patents with COVID & their families for unmet needs, SPC support (social worker, chaplain), SPC consultations via telemedicine, clinical nurse specialist.
20	Cox et al., 2021		USA	RCT	Multi-Centred	Measure impact	3	Integrative, N = 202	ICU Connect app for families to identify PC needs. education, family meeting within 24 hours of identified unmet need.
21	Paré et al., 2021		USA	Mixed methods	SICU	Evaluate families’ experience	N/A	Integrative, N = 5	Embedded practices (GOC conversations within 72 hours of admission, clear documentation of care decisions), joint rounding- multi-disciplinary include. family members, clinician education.
22	Schockett et al., 2021		USA	Pre-Post	MICU	Measure impact	6	Combined, N = 60	Joint rounding -patients with COVID-19, clinician education, structured referral, SPC support.
23	Sinha et al., 2021		USA	Pre-Post	M-S ICU	Measure impact	6	Integrative, N = 350	Joint rounding incl. multi-disciplinary team X 3 days/week, screened all new ICU admissions by asking the question: “Would you be surprised if this patient died during this hospital stay?” If the ans. was No, then a social worker referral was made & GOC conversations were undertaken.
24	Chung et al., 2022		USA	Observational	Multi-Centred	Measure impact	16	Consultative, N = 8991	Screening criteria, SPC referral.
25	Cralley et al., 2022		USA	Pre-Post	SICU	Measure impact	18	Integrative, N = 791	Daily PC GOC considerations in ICU, joint rounding, champion, clinician education, a standardised electronic medical record documentation of PC.
26	Grouls et al., 2022		USA	Retrospective	GICU	Compare impact	3	Consultative, N = 48	Weekly joint rounding interdisciplinary meetings to establish GOC and criteria that would indicate improvement. Weekly family meetings organised by social worker & including SPC.
27	Liu et al., 2022		USA	Observational	MICU	Measure COVID NFR impact	3	Integrative, N = 123	All COVID admissions triggered a joint decision-making conference between primary team/ICU team & SPC on need for SPC referral.
28	Poi et al., 2022		Singapore	Pre-Post	NICU	Measure impact	24	Consultative, N = 388	Screening criteria, weekly joint rounding, weekly interprofessional meetings, structured SPC referrals, clinician education.
29	Shemme et al., 2022		USA	Pre-Post	NICU	Measure impact	24	Consultative, N = 274	Increased SPC & ICU collaborations- 2X weekly joint rounding, referrals were based on perceived PC needs, & a neuro-PC specialist to drive change.
30	Davila et al., 2023		USA	Retrospective	GICU	Measure impact	2	Consultative, N = 22	SPC team had a Spanish speaker on duty/weekly to enhance PC for Spanish-speaking patients in the ICU, joint rounding, screening criteria, weekly handoffs.
31	Helgeson et al., 2023		USA	Prospective RCT	MICU	Measure impact	6	Consultative, N = 91	Screening criteria, SPC consultation within 24 hours of ICU admission.
32	Iguina et al., 2023		USA	Retrospective case–control	M-S ICU	Evaluate screening	3	Combined, N = 388	Screening-criteria, MDTs- discussed, enhancing pain assessment at ICU, encouraging family meeting within 72 hours.
33	Mehta et al., 2023		USA	Pre-Post	MICU	Measure impact	6	Integrative, N = 221	Embedded PC practices, clinician education, offering of spiritual support and identifying medical decision maker. joint rounding 3 weekly and daily multi-disciplinary team meetings (ICU & SPC) with GOC discussions.
34	Rao et al., 2023		India	Qualitative	GICU	Explore ICU-PC strategies	3: 4 workshops, 15 meetings	Combined, N = 13 stakeholders	Family Leaflet, clinician education program 24 modules based on the Education in Palliative and EOLC, weekly 1 hr webinar on PC in ICU, SPC engagement, upskilling ICU clinicians on prognosis & PC assessment skills, referral policies, EOLC policies.
35	Soper et al., 2023		USA	QI project	NICU	Measure impact	12	Consultative, N not provided	ANP in PC embedded into ICU- role incl. symptom management, family support, managing conflicts re goals of care, and disposition planning clinician education. The ANP participated in ICU daily rounding and family meetings.

Review Studies									
Ref.	References		Type		Aim	Systematic Search	Key Findings		
1.	Baker et al., 2015		Review		Explore PC in ICU	No	Emphasised integrating PC to reduce conflicts, clinician burnout, align GOC with patient values. Highlighted screening criteria, daily PC practices, communication strategies, MDT involvement, and clinician proactive education.		
2	Frontera et al., 2015		Review		PC in neuro-ICU	Yes	Identified prognostic tools (GCS, NIHSS, ICH Score) for PC screening. Advocated routine daily PC, ethical considerations, shared decision-making, family communication, early SPC involvement, and MDT collaboration.		
3	O’Connell & Maier, 2016		Review		PC in Trauma ICU	No	Discussed CAPC resources, screening criteria including frailty, integrative models, ethical principles in, combined models, surgeon training, and SPC integration.		
4	Roczen et al., 2016		Systematic Review		PC integration evidence	Yes	Highlighted CAPC checklists, PC education, GOC discussions, symptom management, EOLC protocols, SPC family meetings, and daily ICU PC practices.		
5	Coelho and Yankaskas, 2017, 2017		Review		PC integration	No	Screening for dialysis withdrawal, surprise question. Emphasised structured communication, symptom management, GOC, legal decision-makers, spiritual support, and SPC involvement post-ICU or comfort care.		
6	Harman 2017		Review		PC in ICU	No	Described consultative, integrative, combined models. Discussed screening triggers, EOLC protocols, SPC-led family meetings, MDT involvement, and ICU clinician education.		
7	Adler et al., 2017		Systematic Review		PC in ICU	Yes	Identified screening criteria, ethical and holistic triggers. Emphasised structured communication, shared decision-making, SPC collaboration, education, and inter-disciplinary GOC discussions.		
8	Mun et al., 2017		Review		Identify ICU patients needing PC	Yes	Highlighted trigger criteria for SPC referral, tailored ICU-specific screening, consultative vs integrative models, C&CB quality measures, and clinician-led PC interventions.		
9	Aslakson et al., 2017		Review of reviews		Identify gaps in ICU PC	Yes	Categorised interventions targeting patient/family, clinician, system, and multi-level. Noted SPC consultations, joint rounding, ethics involvement, and structured communication.		
10	Kahveci, 2017 2017		Review		PC integration in ICU	No	Emphasised post-discharge care, structured communication, emotional support, SPC daily rounding, MDT engagement, and clinician education.		
11	Mercadante et al., 2018		Review		PC application in ICU	No	Discussed integrative, consultative, mixed models. Highlighted trigger criteria, daily PC assessments, communication strategies, symptom management, EOLC, and SPC training for ICU clinicians.		
12	Krishnappa et al., 2018		Review		PC for ICU AKI patients	No	Used RIFLE and ASN guidelines for screening. Emphasised family education, early SPC involvement, symptom management, and MDT support.		
13	Rivet et al., 2018		Review		PC in ICU	No	Implemented screening questions in rounds, identified PC needs, facilitated transitions to comfort care, emphasised education, SPC role in symptom management.		
14	Kyeremanteng et al., 2018		Systematic Review		LOS and cost post-PC	Yes	Automated trigger-based SPC consults, reduced LOS and costs, emphasised SPC role in GOC discussions, family meetings, and clinician training.		
15	Crooms and Gelfman, 2020		Review		Frail patients in ICU	No	Consultative and integrative models, frailty-based triggers, psychosocial and ethical considerations, structured communication, online education (ELNEC, EPEC), SPC embedded in ICU MDT.		
16	Creutzfeldt 2021		Review		PC in neuro ICU	No	Early symptom management, proactive care, shared decision-making, MDT collaboration, patient-centred goals.		
17	Aslakson et al., 2021		Review		PC in ICU	No	Early PC assessment, symptom management, prognosis communication, GOC alignment, clinician support, timely SPC involvement.		
18	Metaxa et al., 2021		Systematic Review		Evaluate PC in ICU	Yes	Identified communication interventions, ethics consultations, education, SPC team involvement, structured family meetings, cost reductions, increased DNRs/ADs.		
19	Sady et al., 2021		Review		Neuro ICU PC challenges	No	Four-question checklist, shared decision-making, holistic care, early SPC contact, instructional videos, MDT collaboration, proactive PC approach.		
20	Curtis et al., 2022		Review		PC integration in ICU	No	Screening criteria beyond mortality, ICU culture, teamwork, PACE tool, primary PC skills, SPC training, anticipatory bereavement, spiritual support.		
21	Kim et al., 2022		Review		Cardiac ICU PC principles	No	Screening triggers, GOC focus, communication, emotional support, family-centred decisions, SPC for complex symptoms, clinician education in PC skills.		
22	Gupta et al., 2022		Narrative Review		ICU PC needs	Yes	Screening triggers, symptom management, holistic MDT involvement, education, public awareness, ethical decision-making, SPC collaboration.		
23	Dolmans et al., 2023		Review		Neuro ICU PC	No	Symptom management, grief and spiritual support, family communication, SPC-MDT collaboration, early family meetings, education for ICU clinicians.		
24	Hernández-Zambrano et al., 2024		Integrative Review		Improve EOLC	Yes	Screening criteria, triggers, physical and emotional symptom management, communication tools, structured family meetings, SPC MDT involvement, clinician education.		
25	Wiencek 2024		Review		Current ICU PC	No	Screening triggers, primary PC skills for ICU clinicians, interdisciplinary SPC support, education/training, telemedicine, ICU-SPC collaboration, ethical decision-making.		

*United States of American (USA), Neurological ICU (NICU), Palliative Care (PC), Medical ICU (MICU), Randomised Control Trail (RCT), Specialist Palliative Care (SPC), Care & Communication Bundle (C&CB), General ICU (GICU), Advanced Nurse Practitioner (ANP), length of Stay (LOS), Medical-Surgical ICU (M-S ICU), Intensive Care Units (ICU), End-of-Life Care (EOLC), neuro ICU (NICU), Surgical ICU (SICU), Goals of Care (GOC), Not- For-Resuscitate (NFR), Multi-Discipline Team (MDT), Quality Improvement (QI), Glasgow Coma Scale (GCS), National Institutes of Health Stroke Scale (NIHSS), Intracerebral Haemorrhage Score (ICH), Centre to Advanced Palliative Care (CAPC), Risk, Injury, Failure, Loss, End-stage kidney disease (RIFLE), American Society of Nephrology (ASN), End-of-Life Nursing Education Consortium (ELNEC Consortium), Education in Palliative and End-of-Life Care (EPEC),), Advanced Directives (AD), Probe, Alert, Challenge, Emergency (PACE)

The review studies were published between 2015-2024 (see Table 2). These included four systematic reviews (Adler et al., 2017; Kyeremanteng et al., 2018; Metaxa et al., 2021; Roczen et al., 2016), and one umbrella reviews (Aslakson et al., 2017), with the remaining studies classified as other types of narrative or integrative reviews. Among the non-systematic reviews, five reported conducting a systematic search (Aslakson et al., 2017; Frontera et al., 2015; Gupta et al., 2022; Hernandez-Zambrano et al., 2024; Mun et al., 2017). Collectively, these studies primarily examined the integration of palliative care within ICU settings. However, none offered a systematic analysis of the key characteristics of ICU palliative care studies, highlighting the need for present review to comprehensively map these features.

## 4 Identified themes

This review mapped key characteristics of palliative care integration models within adult ICUs. Analysis revealed four recurring patterns reflecting these characteristics: screening criteria, embedded palliative care practices, education, and specialist palliative care collaboration. Table 3 presents a representative thematic analysis.

**Table 3:** Representative Thematic Analysis.

THEME 1: TRIGGER CRITERION		
*Sub-themes*	*Codes*	*Representative References*
Varied role Screening responsibility	Conducted by ICU nurses, embedded SPC teams, multidisciplinary teams, automated systems, Research team, Unknown, Automatic	Adler et al., 2017; Carson et al., 2016; Henderson et al., 2017; Iguina et al., 2023; Krishnappa et al., 2018; Ma et al., 2019; Martz et al., 2020; Mun et al., 2018; O'Mahony et al., 2017; Poi et al., 2022; Schoenherr et al., 2020
Early Identification of Palliative Needs in ICUs Using Screening Tools & Triggers	Systematic screening tools (Predefined triggers, Prognostic / frailty scales, ICU-specific tools, the Needs, Existential concerns, Symptoms Tools, Therapeutic interactions and the Palliative Care Needs Screening Tool, frailty/prognostic scales, EMR integration, Referral pathways, Timings), Clinician-led interventions, Mortality / risk-based screening, Transitional care triggers, Holistic / psychosocial triggers, Ethical triggers	Davila et al., 2023; Gupta et al., 2022, Iguina et al., 2023; Martz et al., 2020; Poi et al., 2022; Rivet et al., 2018; Zalenski et al., 2017
Workflow Integration of Palliative Care Screening	Screening during rounding by MDT, Nursing-Driven Screening Integration, Positive Screens Trigger Automatic Specialist Referral, Digital Workflow Integration, Physician Oversight, Administrative, Limited Integration into Practice	Anderson et al., 2017; Constantine et al., 2016; Cox et al., 2018; Creutzfeldt et al., 2015; Kyeremanteng et al., 2020; Liu et al., 2022; Martz et al., 2020; McCarroll, 2018; Schoenherr et al., 2020; Metaxa et al., 2021
Needs Assessment Strategies	Screening questions or scales (e.g. “*Would you be surprised if this patient died during this hospital stay*?”), NEST tool, PNST	Constantine et al., 2016; Creutzfeldt et al., 2015; Cox et al., 2018, 2021; Sinha et al., 2021; Schoenherr et al., 2020
Validation & feasibility	Derived from expert consensus or literature; formal validation mostly absent; feasibility assessed via usability, adherence, acceptability; clinical relevance; Pilot-tested	Braus et al., 2016; Henderson et al., 2017; Jenko et al., 2015; Mun et al., 2018; Zalenski et al., 2017
Implementation challenges / barriers	Heterogeneity in who screens, unclear workflows, time pressures, competing priorities, SPC stigma, resistance to referrals; limited organisational support, Limited adoption; SPC stigma	McCarroll, 2018; Cox et al., 2018; Iguina et al., 2023; Wiencek, 2024; Zalenski et al., 2017

THEME 2: EMBEDDED PRACTICES		
Subthemes	Codes	References
Workflow Integration	ICU teams assess symptoms, initiate goals-of-care discussions, document advance directives, and engage families to align care with patient values	Baker et al., 2015; Carson et al., 2016; Frontera et al., 2015; Mehta et al., 2023; Roa et al., 2023; Soper et al., 2023
	Care & Communication Bundle applied across ICU days 1, 3, 5: pain assessment, decision-maker identification, family support; improved documentation, relational aspects variable	Constantine et al., 2016; Mun et al., 2016, 2017; Vuong et al., 2019
Bridging Roles	Palliative care nurse practitioners join rounds; ICU nurse champions identify unmet needs, facilitate communication, and link specialist care to bedside practice	Davila et al., 2023; Mehta et al., 2023; Poi et al., 2022; Soper et al., 2023
Stakeholder Engagement	ICU leadership and staff adapt palliative practices to local context	Anderson et al., 2017; Cralley et al., 2022; Iguina et al., 2023; Gupta et al., 2022
Challenges	Variable adoption, limited policy support, resource constraints, workflow complexity, curative culture, misconceptions	Aslakson et al., 2017; Gupta et al., 2022; Kyeremanteng et al., 2018; Iguina et al., 2023
Relational Competence	Integration relies on visible role models, interprofessional communication, and ethical alignment; ensures patient values are respected	Baker et al., 2015; Roczen et al., 2016; Frontera et al., 2015; O’Connell & Maier, 2016
Continuous training	Ongoing education strengthens clinician confidence, ethical decision-making, communication, and real-time application of palliative principles	Jenko et al., 2015; Mehta et al., 2023

THEME 3: EDUCATION		
Sub-theme	Codes	References
Education Resources	CAPC, EPEC-ICU, ELNEC, VitalTalk, C-3 program	Anderson et al., 2017; McCarroll, 2018
Practical Application	PPSv2, PNST, bedside skill practice	Creutzfeldt et al., 2015; Jenko et al., 2015; Soper et al., 2023
Diverse Teaching Formats	Online modules, workshops, role modelling, instructional videos, academic detailing	Constantine et al., 2016; McCarroll, 2018
Communication Skills Training	Goal-of-Care/End-of-Life workshops, NURSE framework, PACE tool, Best Case/Worst Case	Carson et al., 2016; Curtis et al., 2022
Core Content & Skills	Family support, symptom management, shared decision-making, ethical principles	Roczen et al., 2016; Poi et al., 2022
Family Education	Informational materials, VALUE format, coaching families	Paré et al., 2021; Cox et al., 2018
Targeted Education	ICU-specific, neurologist/SPC training, MD/PhD programs	Mehta et al., 2023; Anderson et al., 2017
Nurse-Led Education	Bedside education, awareness raising	Creutzfeldt et al., 2015
Ethical & Relational Aspects	Ethics consultations, moral decision-making, addressing disparities	Wiencek, 2024
Enhance Acceptability & Engagement	Reducing stigma, cultural barriers, reframing PC as quality-of-life	Anderson et al., 2017; Metaxa et al., 2021
Challenges	Discipline-specific, episodic, resource-intensive delivery, inconsistent approaches across ICUs, focused on individual skills, resistance, competing demands, financial constraints	Anderson et al., 2017; Baker et al., 2015; Constantine et al., 2016; Gupta et al., 2022; Jenko et al., 2015; McCarroll, 2018; Metaxa et al., 2021

THEME 4: Increased Specialist Collaboration		
Subthemes	Codes	References
**Joint ICU–SPC Rounding**	Daily or weekly interdisciplinary rounds; proactive co-management; teleconference or bedside participation; inclusion of ethics, geriatrics, and social work perspectives; interprofessional morning meetings.	Kyeremanteng et al., 2020; Liu et al., 2022; Ma et al., 2019; Poi et al., 2022; Schockett et al., 2021; Shemme et al., 2022; Sinha et al., 2021; Vig, 2019; Braus et al., 2016; Hernández-Zambrano et al., 2024
**Operational Integration**	Early/automatic referral; structured protocols and algorithms embedded into admission pathways; hospice-supported or step-down palliative pathways; routine consultation within 48 hrs of admission; proactive case-finding by specialist nurses; technology-supported coordination (e.g., PCplanner app)	Cox et al., 2018; Grouls et al., 2022; Henderson et al., 2017; Ma et al., 2019; Morris et al., 2021; Shemme et al., 2022; Metaxa et al., 2021; O’Mahony et al., 2017; Zalenski et al., 2017
**Family Engagement and Decision Support**	SPC-supported family meetings; shared decision-making; structured communication tools; emotional support and bereavement resources.	Carson et al., 2016; Ma et al., 2019; Martz et al., 2020; Poi et al., 2022; Shemme et al., 2022; Kim et al., 2022
**Symptom and Supportive Care Management**	Advanced symptom assessment and management; emotional, social, and spiritual interventions; early goals-of-care discussions; protocols for terminal extubation and discharge.	Chung et al., 2022; Henderson et al., 2017; Poi et al., 2022; Shemme et al., 2022; Ma et al., 2019; Mun et al., 2016; Dolmans et al., 2023
**Operational Processes / Workflow Routines**	Embedded referral triggers; feedback loops; team huddles; communication coaching; use of champions and checklists; integration of SPC consultation into inpatient service; technology-supported coordination (e.g., PCplanner app); joint GOC discussions and multidisciplinary meetings; Excessive dependence on specialist may lead to fragmented care and reduce motivation.	Chung et al., 2022; Cralley et al., 2022; Cox et al., 2018; Davila et al., 2023; Frontera et al., 2015; Grouls et al., 2022; Liu et al., 2022; Ma et al., 2019; Paré et al., 2021; Poi et al., 2022; Schockett et al., 2021
**Multidisciplinary Team Collaboration**	Inclusion of chaplains, psychologists, social workers, and physicians; enhanced communication; interprofessional working groups;	Chung et al., 2022; Cralley et al., 2022; Shemme et al., 2022; Braus et al., 2016; Poi et al., 2022; Shemme et al., 2022
**Care Continuity**	Continued SPC follow-up post-ICU to support recovery, step-down care, or end-of-life transition.	Poi et al., 2022; Shemme et al., 2022

*Note. Sub-themes represent theme strategies identified across the included research & review studies. A full table with detailed examples and codes is available in Supplement file S2. *Intensive Care Units (ICU), Specialist Palliative Care (SPC), Needs, Existential concerns, Symptoms Tools (NEST), the Palliative Care Needs Screening Tool (PNST), Electrical Medical Records (EMR), Multidisciplinary Team (MDT), Centre of Advanced Palliative Care (CPAP), Education in Palliative and End-of-Life Care (EPEC), End-of-Life Nursing Education Consortium (ELNEC), Palliative Performance Scale version 2 (PPSv2), Name, Understand, Respect, Support, Explore (NURSE), Value, Acknowledge, Listen, Understand, Elicit (VALUE), Goals of Care (GOC)

### 4.1 Theme 1: Screening Criteria

Analysis identified screening criteria as a defining feature of palliative care integration, providing a structured approach to identify patients with complex needs. Screening tools were employed in 23 of the 35 research studies, while 20 of the 25 review studies discussed them (see Table 2). Mun et al. (2016) and Mun et al. (2018) applied identical screening criteria. Across this evidence, screening was typically undertaken within 48–72 hours of ICU admission, for timely palliative care referrals (Adler et al., 2017; Davila et al., 2023; Mahta et al., 2023). Most studies adopted trigger-based checklists often adapted from the Centre to Advance Palliative Care (CAPC) framework or ICU specific tools like APACHE II, and the Rothman Index (Henderson et al., 2017; Ma et al., 2019; Martz et al., 2020). Triggers varied widely, covering disease severity, frailty, healthcare utilisation, and holistic concerns (see Table 4). While this variability allowed for local adaptation, it limited comparability across settings.

**Table 4:** Screening Criteria: Trigger types.

	Disease severity and trajectory	Acute physiological instability	Healthcare utilisation and course	Frailty and functional decline	Psychosocial, ethical, or communication concerns
Creutzfeldt et al., 2015	*		*	*	
Jenko et al., 2015				*	
Constantine et al., 2016	*	*	*	*	
Braus et al., 2016	*		*	*	
Carson et al., 2016			*	*	
Anderson et al., 2017	*	*		*	
Henderson et al., 2017	*		*		
O'Mahony et al., 2017	*		*		
Zalenski et al., 2017	*	*		*	
Mun et al., 2017, 2018	*		*	*	*
Cox et al., 2018	*	*	*	*	
Mc Carroll, 2018	*	*	*	*	*
Ma et al., 2019	*	*	*	*	
Martz et al., 2020	*	*	*	*	*
Schoenherr et al., 2020		*		*	*
Sinha et al., 2021					*
Chung et al., 2022	*	*		*	
Liu et al., 2022	*			*	
Poi et al. 2022	*	*		*	
Davila et al., 2023	*		*	*	
Helgeson et al., 2023	*	*		*	
Iguina et al., 2023	*	*	*	*	*

⁎ A full table with detailed triggers is available in Supplement file S3.

Earlier studies primarily focused physical triggers whereas more recent studies increasingly incorporate psychosocial indicators such as caregiver burden and family distress (Cox et al., 2021; Creutzfeldt et al., 2015; Curtis et al., 2022). This reflects a gradual shift toward holistic assessment approaches. Despite this progress, illness severity remained the predominant trigger for specialist referral across studies, reinforcing perceptions of palliative care as end-of-life support rather than an integrated component of critical care (Cox et al., 2021; Ma et al., 2019). Across the included studies, responsibility for screening varied considerably. In several studies, nurses conducting daily screening, while others integrated screening into multidisciplinary rounds (Poi et al., 2022; Davila et al., 2023; Iguina et al., 2023). In some cases, screening was performed by researchers (see Table 3, theme 1), further highlighting inconsistencies in how screening was operationalised. Several studies also reported clinician uncertainty regarding which triggers to apply and how to use them consistently (McCarroll, 2018; Zalenski et al., 2017). Despite this variability, most studies utilised checklist formats to guide referrals based on patients’ need rather than provider discretion (Helgeson et al., 2023; O’Connell & Maier, 2016; Sinha et al., 2021). However, checklist approaches risk becoming tick-box exercises, constraining deeper exploration of patient goals and evolving needs.

From this evidence base most tools were developed through expert consensus or adapted from existing literature, with few undergoing formal validations for screening accuracy (Helgeson et al., 2023; Mun et al., 2018; Zalenski et al., 2017). Within some studies pilot testing with ICU clinicians demonstrated feasibility and integration into clinical workflows; yet inconsistent validation and reliance on local champions or short-term initiatives limited scalability (Mun et al., 2018; Cox et al., 2021; Martz et al., 2020). Across studies, adherence to screening was generally moderate, however integration into routine practice remained uneven. This was hindered by time constraints, competing clinical demands, and unclear referral pathways. Implementation was often confined to individual initiatives, reflecting limited organisational support for sustained integration and scalability.

Although screening frequently identified palliative care needs within most studies, specialist involvement did not consistently follow (Chung et al., 2022; Cox et al., 2018; Harman et al., 2017; Zalenski et al., 2017). This raises questions about whether ICU teams addressed these needs using primary palliative care skills, though few studies reported follow-through at the ICU level. Referral decisions typically rested with ICU consultants, and resistance to specialist involvement occasionally prevented eligible patients from receiving appropriate care, leaving real-world impact of screening uncertain (Gupta et al., 2022; Kyeremanteng et al., 2018).

Overall, across studies screening criteria serve as a foundational mechanism for integrating palliative care into ICU practice, guiding both specialist referrals and clinician-led interventions to promote equitable and timely care. However, variable triggers, inconsistent application, and limited validation constrain their effectiveness. These findings suggest that while screening is essential for identifying early palliative needs, it is insufficient in isolation and should be complemented with embedding primary palliative care principles into everyday ICU practice, as explored in the next theme.

### 4.2 Theme 2: Embedded Practices

Embedded primary palliative care practices emerged as a defining feature of ICU-level integration within daily clinical workflows. Most reviewed studies highlighted the importance of routine practices, particularly in contexts with limited access to specialist palliative care, to ensure patient comfort, emotional support, and alignment of care with patient centred values, see Table 3, theme 2, (Creutzfeldt, 2021; Harman, 2017). Across studies, ICU teams consistently assessed symptoms, initiated early goals-of-care discussions, documented advance directives, and proactively engaged families to align treatment with individual values (Mehta et al., 2023; Roa et al., 2023; Soper et al., 2023). Despite these efforts, several limitations were identified, included competing clinical demands, short intervention durations, variability in intervention design, and the predominance of studies from high-income countries, factors which constrain the generalisability of findings.

Across research studies dedicated roles such as palliative care nurse practitioners and ICU nurse champions, were instrumental in: bridging specialist and bedside care, identifying unmet needs and facilitating these practices (Davila et al., 2023; Mehta et al., 2023; Soper et al., 2023). While their effectiveness was influenced by local resources, training, and ICU culture, these roles enabled ICU teams to manage core palliative needs and reserve specialist input for complex cases, thereby improving early communication and care coordination.

Across studies, engagement of key stakeholders, including leaders and frontline clinicians, emerged as a central strategy for tailoring palliative care practices to the specific context and workflow of individual ICUs (Iguina et al., 2023; Mehta et al., 2023; Soper et al., 2023). Nevertheless, implementation remained heterogeneous, with some units demonstrating strong adoption and others facing challenges reflecting ongoing to barriers to system-wide integration (Anderson et al., 2017; Cralley et al., 2022). These barriers included gaps in policy, lack of tailored interventions, inconsistent stakeholder engagement and persistent misconceptions equating palliative care with end-of-life care. Resource constraints, and cultural resistance further compounded these challenges.

Within the included studies, integrating palliative care principles into daily workflows, such as ICU rounds and electronic medical records, supported systematic identification of patients with palliative needs (Mun et al., 2018; Soper et al., 2023). Tools like the Palliative Performance Scale and structured screening criteria consistently facilitated timely interventions (Iguina et al., 2023; Jenko et al., 2015; McCarroll, 2018). However, limited palliative care training undermined the consistent application of these practices. Time-sequenced pathways, like the Care and Communication Bundle, emerged across studies as one of the most structured and evidence-based approaches to embedding primary palliative care principles within ICU practice (Constantine et al., 2016; Mun et al., 2017; Vuong et al., 2019). Implemented across days one, three, and five, this bundle integrated essential tasks into routine ICU care, including identifying decision-makers, documenting advance directives, assessing pain, and supporting families (see Table 5).

**Table 5:** Care and Communication Bundle.

Domains	Measures
**ICU DAY ONE**	
Patient/family-centred decision-making	Determination of medical decision-maker
Patient/family-centred decision-making	Determination of DNF code status
Communication and health literacy	Family information leaflet
Symptom management	Pain assessment and management
**ICU DAY THREE**	
Emotional and social support for patient/families	Social work support offered to patient/families
Spiritual support of patient/families	Social work support offered to patient/families
**ICU DAY FIVE**	
Communication	Communication/Interdisciplinary family meeting

*(Vuong et al. 2019, pp2)

This process-driven framework aligns with the US national palliative care quality standards (Mun et al., 2017). However, its application was limited, with only four studies reporting its use, representing just three unique implementations (Constantine et al., 2016; Mun et al., 2016, 2017; Vuong et al., 2019). This limited adoption highlights its uneven integration into real-world ICU settings. The most consistent improvements were observed in procedural documentation, such as identifying decision-makers and completing advance directives. However, more relational aspects, like engaging in meaningful conversations with families or addressing spiritual needs, showed more variable outcomes with no significant improved noted. These findings highlight persistent ongoing challenges in embedding the full scope of palliative care into ICU routines.

While bundles and checklists improved procedural consistency, they risked reducing the complex, human dimensions of care to measurable targets (Baker et al., 2015; Roczen et al., 2016). Frontera et al. (2015) and O’Connell and Maier (2016) further emphasised that successful integration depends not only on protocols, but also on ethical alignment, interprofessional communication, and visible role modelling. Collectively, these findings suggest that sustainable palliative care integration requires both structural frameworks and relational competence, reinforced through continuous education, leadership engagement, and locally adapted implementation strategies. Ultimately the effectiveness of embedded models hinges on teams’ capacity to interpret and enact palliative principles in real time, positioning education not as an adjunct, but as a central mechanism for maintaining alignment with core values, as explored in the following theme.

### 4.3 Theme 3: Education

Education emerged as a key characteristic in the integration of palliative care within ICU settings (see Table 3, theme 4). Education addressed critical knowledge gaps, corrected misconceptions, and enhanced clinicians’ competence in deliver primary palliative care in the ICU (Anderson et al., 2017; Metaxa et al., 2021). Of the 35 research studies reviewed, 20 incorporated education as a core component, while 20 of 25 review studies identified persistent deficits in clinicians’ palliative knowledge and skills (Metaxa et al., 2021; Wiencek, 2024).

Across both research and review studies, a consistent pattern emerged: limited understanding of palliative care among ICU clinicians remains a major barrier to early adoption (see Table 3). Many ICU professionals continue to associate palliative care solely with end-of-life care, reinforcing a false dichotomy between cure-focused and comfort-focused approaches (Mercadante et al., 2018; Aslakson et al., 2017). This misconception delays goals-of-care discussions and hinders timely identification of patients who could benefit from concurrent palliative input. Across the research studies targeted, context-specific education, ranging from six-month structured nurse modules to interdisciplinary workshops and bedside mentoring, was shown to improve ICU clinicians’ knowledge, confidence, and communication skills. These interventions corrected misconceptions and strengthened readiness for sustained palliative care integration in ICU (Anderson et al., 2017; Constantine et al., 2016; McCarroll, 2018).

Within these studies, education was most effective when embedded within daily ICU workflows rather than delivered as isolated training events. A predominant focus on individual skills, rather than team-based and system level approaches, limited its potential to drive sustained behavioural and cultural change (Table 3, theme 4). Nurse champions and palliative liaisons played a pivotal bridging role, translating theoretical learning into bedside practice. Their presence facilitated identifying unmet needs, modelled effective communication, and ensured specialist input was reserved for complex cases. Yet, the success of these roles was highly dependent on local culture, leadership engagement, and access to ongoing education.

Gaps in communication particularly around patient values and end-of-life decisions were repeatedly identified, reinforcing the need for structured communication training (Dolmans et al., 2023; Hernandez-Zambrano et al., 2024). Recommended content included goals-of-care discussions, palliative needs assessment, and end-of-life communication skills, enabling ICU clinicians to provide primary palliative care while reserving specialist input for complex cases (Anderson et al., 2017; Curtis et al., 2022; Roczen et al., 2016). However more nuanced aspects such as meaningful conversations and spiritual support remained underdeveloped (Mun et al., 2017; Vuong et al., 2019). Across studies few interventions included education practical guidance on implementation, revealing a disconnect between strategy development and real-world application (Creutzfeldt et al., 2015; Soper et al., 2023).

Similarly, family education was rarely incorporated (Cox et al., 2021; Mun et al., 2017), despite evidence that clinicians frequently avoid palliative discussions due to concerns about being perceived as abandoning curative intent (Frontera et al., 2015). When included, family education typically involved providing informational materials or structured meetings rather than ongoing engagement, highlighting a need for more robust strategies to empower families to participate actively in decision-making. Few interventions targeted education of specialist palliative care teams, despite the benefits of reciprocal training for ICU collaboration (Anderson et al., 2017; Mehta et al., 2023).

In summary, education is foundational, yet multifaceted. It strengthens clinician capability, supports patient-centred practice, and fosters integration, but its impact depends on sustainable, system-aware implementation that goes beyond individual skill development or competence. Building clinicians’ knowledge and confidence through education naturally sets the stage for the next key theme: increased collaboration with specialist palliative care teams, where shared understanding and coordinated practice further enhance integration in the ICU.

### 4.4 Theme 4: Specialist Palliative Care Collaborations

Specialised collaboration emerged as a central feature in models for integrating palliative care into ICU settings discussed in 25 of 35 research studies and all included reviews (see Table 2). Collaboration varied depending on how deeply specialist palliative care teams were embedded within ICU workflows. Integrated and hybrid models placed specialist clinicians alongside ICU clinicians during daily rounds, shared decision-making, and family discussions, promising consistency and earlier intervention (Liu et al., 2022; Rao et al., 2023; Schockett et al., 2021). In contrast, consultative models relied on demanded specialist input, which optimised resources but left primary palliative responsibility with ICU teams (Gupta et al., 2022; Ma et al., 2019).

From this evidence base, fully integrated models demonstrated stronger effects on communication and goal-concordant care but required sustained specialist time and institutional commitment, limiting scalability (Liu et al., 2022; Mehta et al., 2023). Consultative approaches provided greater flexibility but often led to delayed referrals and fragmented care, reinforcing dependence on specialist palliative care teams and limiting ICU clinicians’ palliative care competencies (Gupta et al., 2022; Mun et al., 2018). Hybrid models which embedded specialist expertise while empowering ICU clinician to deliver primary palliative principles appeared the most practical and sustainable balance (Iguina et al., 2023; Rao et al., 2023; Schockett et al., 2021).

Across models, specialist involvement consistently enhanced communication, supported decision-making, and aligned treatment with patient values (Mehta et al., 2023; Soper et al., 2023). However, benefits were inconsistent, and few studies employed standardised outcome measures, limiting reproducibility and scalability (Carson et al., 2016; Ma et al., 2019). Integration was most effective where formalised processes, clear role delineation, and organisational support were present (Liu et al., 2022; Rao et al., 2023). Ad hoc or consultative-only involvement had limited operational impact (Cox et al., 2021; Mun et al., 2018). Multidisciplinary engagement including nurses, social workers, chaplains, and allied professionals, were universally reported, suggesting that relational collaboration complements structural integration. However, the essential component of such collaboration remains unclear.

Analysis showed that screening and referral mechanisms such as automated triggers, morning checklists, or ICU-wide screening facilitated early specialist involvement and shifted practice from reactive consultation to systematic integration (Kyeremanteng et al., 2018; Davila et al., 2023). These mechanisms promoted earlier identification, symptom control, and shared decision-making (Carson et al., 2016; Martz et al., 2020). Despite these gains, implementation was constrained by workflow pressures, entrenched professional norms, and ambiguous role boundaries constraining implementation (Iguina et al., 2023; Rao et al., 2023). Family engagement emerged as a consistent focus with specialist-led meetings facilitating earlier involvement, improving communication, and supporting confident, goal-concordant decisions (Cox et sl., 2018; Helgeson et al., 2023). Analysis showed collaboration with specialist palliative care improved management of refractory symptoms and supported anticipatory planning and emotional support for patients, families, and staff (Cralley et al., 2022; Shemme et al., 2022).

Several studies cautioned that overreliance on specialist teams may fragment care and impede development of ICU clinicians’ palliative competencies, skills essential for sustaining integration (Gupta et al., 2022; Mehta et al., 2023). Effective collaboration also required specialist teams to understand ICU culture, overcome resistance and clarify their roles (Anderson et al., 2017; Mehta et al., 2023). In some cases, physician reluctance to refer patients, reflected persistent attitudinal and cultural barriers (Carson et al., 2016; Creutzfeldt et al., 2015).

Post-ICU continuity through goals-of-care discussions, advance directives, and follow-up education helped extend collaboration beyond the ICU and supported consistency of care across the patient trajectory (Cralley et al., 2022; Liu et al., 2022; Rao et al., 2023). Although pre–post studies frequently reported enhanced teamwork and collaboration, the overall evidence base remains limited, with few RCTs and a predominance of studies from the United States studies, thereby restricting generalisability.

## 5 Discussion

We identified four key themes that characterise palliative care integration models in adult ICUs across the included studies. These were screening criteria, embedded palliative care practices, education, and specialist palliative care collaboration. Screening tools and embedded practices such as daily assessments provided structured, early mechanisms to identify and address needs, promoting more equitable access to palliative care (Helgeson et al., 2023; Iguina et al., 2023). These approaches reflect international standards that advocate for early palliative care alongside active treatment, regardless of prognosis (Payne et al. 2022; WHO, 2020). However, implementation frequently relied on individual champions rather than sustained organisational commitment (Davila et al., 2023; Soper et al., 2023). While screening alone did not consistently lead to specialist referrals due to cultural barriers and variability in who performed the assessments. This shows the need for clear role delineation and integrated workflows in future models.

None of the included research studies reported validating their screening tools, most relied on expert consensus or existing literature (Helgeson et al., 2023; Mun et al., 2018; Zalenski et al., 2017). Without validation, the risk of under- or over-referral can comprise the quality and consistency of care. While automated screening can facilitate early specialist involvement, it may also diminish ICU team autonomy (Cox et al., 2018). This highlights the need to balance specialist palliative care input with ICU-delivered primary palliative care.

Across the research studies, screening criteria predominantly focused on disease severity and functional decline. This contrasts with the broader literature, which increasingly supports holistic, needs-based triggers rather than triggers based solely on disease status (Adler et al., 2017). By contrast, several included reviews advocated a shift away from mortality-based criteria (Curtis et al., 2022; Creutzfeldt, 2021). Balancing high-risk triggers with person-centred indicators may help challenge perceptions that palliative care is limited to end-of-life scenarios (Curtis et al., 2022; Payne et al., 2022). Future research should prioritise validating screening tools, integrate holistic triggers, and translating needs into timely bedside action.

This analysis highlighted inconsistencies in the real-world application of embedded practices. Tools such as the Care and Communication Bundle provide a structured framework for integrating palliative principles into daily routines. However, their implementation has been largely confined to the United States, raising questions about adaptability and long-term sustainability in diverse healthcare environments. Future studies should evaluate how these frameworks perform across varied ICU contexts, including resource-constrained settings to assess feasibility, cultural relevance and impact.

Collectively, these findings highlight that education is central to sustainable integration. Across the included studies, targeted education enhanced clinicians’ confidence in communication, ethical decision-making, and goals-of-care discussions (McCarroll, 2018; Metaxa et al., 2021). Educational approaches commonly combined theoretical learning with bedside coaching, strengthening interdisciplinary teamwork and supporting timely, person-centred decision-making (Kim et al., 2022). Together, these findings suggest that education helped address stigma by reframing palliative care from a reactive, end-stage measure to an anticipatory, patient-centred approach that complements curative treatment.

Although not specific to ICU settings, this approach aligns with international guidelines advocating early, proactive palliative care as a fundamental right rather than a last resort (EAPC, 2019). Equipping ICU clinicians with primary palliative care skills is essential, particularly in settings where specialist availability is limited. These findings reflect global initiatives that call for clinicians frequently involved in end-of-life care such as ICU clinicians, to develop advanced primary palliative competencies (WHO, 2020; EAPC, 2019). Strengthening primary palliative skills within ICU teams is critical to ensuring holistic, safe, and consistent care.

Evidence across this review identified collaboration between ICU and specialist palliative care teams as a key feature of successful palliative care integration (Davila et al., 2023; Mehta et al., 2023; Soper et al., 2023). Similar trends have been observed in heart failure, dementia, and chronic obstructive pulmonary disease, where early palliative care involvement improved quality of life and reduced healthcare costs (Hepgul et al., 2018; Scheerens et al., 2018). Analysis revealed three collaboration levels:

1. Fully embedded models integrate specialist teams into ICU workflows through daily rounds, shared decision-making, and family meetings, enhancing communication, symptom management, and goal-concordant care (Liu et al., 2022).
2. Consultative models have the greatest impact. However, they require sustained specialist time, limiting scalability (Gupta et al., 2022).
3. Hybrid models combine specialist expertise with developing clinicians’ primary palliative skills, offering a balanced and sustainable approach to integration (Rao et al., 2023).

This reflects wider debates in global palliative policy, where international organisations emphasise that early integration should not rely solely on specialist teams but should build palliative competencies among clinicians, including ICU clinicians (WHO, 2015; EAPC, 2019). Taken together, these findings suggest that hybrid models may represent the most policy-aligned and scalable approach to palliative care integration. Nevertheless, these findings suggest that specialist integration in ICUs remains inconsistent, as stigma, workflow demands, unclear role boundaries, and entrenched cultural norms constrain adoption. Few included studies evaluated post-ICU continuity or long-term patients’ outcomes (Poi et al., 2022). Future research should clearly define specialist palliative care roles in ICUs, address cultural misunderstandings, and demonstrate the value of ICU–palliative care collaboration. Evaluating post-ICU palliative care continuity and long-term outcomes is essential to ensure that specialist integration has a sustained and meaningful impact.

More broadly, the challenges identified across themes mirror wider patterns in healthcare research, where promising interventions often struggle to progress beyond early pilot phases (Peters et al., 2020). Organisational and policy contexts play a central role in shaping how palliative care integration is integrated within ICU settings. The themes in this review suggest that successful integration depends upon organisational support for structured screening processes, embedding palliative care practices into routine ICU workflows, and ensuring access to education and specialist palliative care expertise. Institutional policies, staffing levels, and the availability of specialist palliative care teams directly affect the feasibility and sustainability of these approaches. National policy frameworks and guidance on palliative and end-of-life care further influence how integration models are operationalised across healthcare systems. Ultimately, sustainable adoption requires supportive organisational structures, clear processes, and contextual adaptation (Damschroder et al., 2009; Woodward et al., 2021). Strong organisational commitment is essential to ensure consistent application of screening, embedded practice, education, and collaborative care across systems. Future research should address structural and cultural barriers, evaluate long-term sustainability, and identify which integration components are universally effective and which require local adaptation. Integration efforts must balance fidelity with flexibility, adapting to local culture, resources, and interprofessional dynamics.

The dominating non-experimental designs within this study reflects the practical and ethical challenges of evaluating complex interventions in real-world practice. It also suggests that research on integrating palliative care in ICUs remains largely implementation focused. Although these studies provide valuable insights into feasibility and clinical practice change, they also highlight the need for more robust evaluation.

The predominance of United States based evidence limits understanding of how integration models function across different health systems. Differences in organisational structures, workforce availability, and legal frameworks may shape the feasibility and sustainability of palliative care integration in other ICU settings. Broader international research is therefore needed to identify context-sensitive strategies and inform globally relevant models of palliative care integration.

## 6 Limitations

Although this review provides a systematic synthesis of key characteristics of ICU palliative care integration models, several limitations may affect the interpretation and generalisability of the finding. First, most included studies were United States-based. This may limit the generalisability of the findings to other healthcare systems where organisational structures, workforce availability, and policy frameworks differ. In addition, regional variations in how palliative and end-of-life care are conceptualised and implemented may influence how interventions are described and evaluated. In the US, for instance, palliative care is frequently integrated with curative efforts, while in many European nations, it is more closely associated with end-of-life care. Such differences in vocabulary and cultural understanding may have influenced both the scope and focus of included studies. Future research should explore these regional variations to clarify how palliative care is understood and delivered globally within ICU settings.

Second the heterogeneity of study designs, populations and interventions, limits direct comparison across studies. The majority were observational and single centre which constrains the generalisability of the findings. Additionally, nearly all studies originated in high-income countries, mostly the United States, and therefore may not reflect ICU practices in Europe or low- and middle-income settings, where staffing, resources, and organisational structures vary. Few studies examined long-term outcomes, focusing instead on short-term measures. Finally, consistent with scoping review methodology, we did not undertake a methodological quality appraisal of the included studies. As such, the findings should be interpreted as a mapping of existing approaches rather than an assessment of intervention effectiveness. Future research needs to investigate how the key characteristics identified in this review can be adapted and implemented across diverse ICUs, and cultures with attention to feasibility, sustainability, and local context.

## 7 Conclusion

This review highlights that successful ICU palliative care integration requires more than implementing screening tools. It depends on a collection of interrelated characteristics, embedded practices, continuous education, and collaborative specialist involvement, all supported by system-level adaptability that aligns with ICU workflows and culture. Sustainable integration demands attention to organisational structures, interdisciplinary collaboration, and policy alignment to ensure that palliative care is delivered in a timely, equitable, and person-centred manner. Sustainable integration demands attention to organisational structures, interdisciplinary collaboration, and policy alignment to ensure that palliative care is delivered in a timely, equitable, and person-centred manner.

## Acknowledgments

Dr. Allister Nicol, Consultant in Intensive Care Medicine & Professor of Critical Care, Dr Donal Ryan, Consultant in Intensive Care Medicine, St Vincent’s University Hospital (SVUH), Prof. Ian Callanan, Group Head of Clinical Audit, SVUH and Jessica Eustace-Cook, Academic Librarian, School of Nursing and Midwifery, Trinity College Dublin.

## Funding

This work was supported by the Irish Research Council, (GOIPG/2020/372, 2020). The funding body had no participation in preparing or writing this article.

## CRediT authorship contribution statement

**Yvonne King:** Writing – review & editing, Writing – original draft, Funding acquisition, Formal analysis, Conceptualization. **Peter May:** Supervision, Writing – review & editing. **Barbara Whyte:** Investigation, Data curation, Writing – review & editing. **Eóin Tiernan:** Writing – review & editing. **Anne-Marie Brady:** Supervision, Writing – review & editing.

## Declaration of competing interest

The authors declare the following financial interests/personal relationships which may be considered as potential competing interests: Yvonne King (Muldowney) reports was provided by Trinity College Dublin. If there are other authors, they declare that they have no known competing financial interests or personal relationships that could have appeared to influence the work reported in this paper.

## Appendix Supplementary materials

Image, application 1

Image, application 2

Image, application 3

Image, application 4

Image, application 5
