# Supplementary material for: Key characteristics of palliative care integration in intensive care units (ICUs): A scoping review
Source: Int J Nurs Stud Adv. 2026 Apr 8;10:100535. doi: 10.1016/j.ijnsa.2026.100535 (PMC13091130; doi:10.1016/j.ijnsa.2026.100535)
Supplement: Supplementary file 1 [file mmc1.docx]

**SUPPLEMENT FILE S3**

Details of screening criteria

| **Author** | **Aim of screening criteria** | **Format**  **of tool** | **Reliability**  **&**  **validity** | **No. of criteria items** | **Tailored to ICU** | **Deprived from** | **Decision for SPC referral** | **Outcome** |
| --- | --- | --- | --- | --- | --- | --- | --- | --- |
| Jenko et al., 2015 | To identify those who may benefit from timelier SPC | Checklist | Yes- in other settings | 11 | No | Modification of the Karnofsky Performance Scale in 1996  & from CAPC | SPC & ICU Team | Positive- increase in SPC consultation (h110% increase in SPC referrals) & positive response to its use by ICU nurses |
| Creutzfeldt et al., 2015 | To identify those who may benefit from timelier SPC | Questions | No but -Pilot tested | 4 | N/A | Literature review and expert opinion | ICU physician/ Intensivist | Positive-increase in PC consultations-Trend =more PC consultations (p=0.056) & more likely to have a family conference documented |
| Braus et al. 2016 | To identify those who may benefit from timelier SPC | Checklist | No | 8 | No | As per Norton, et. al. 2007 | PC & ICU Team | Positive-more likely SPC principles implemented in patient care & < hospital LOS. No different in ICU LOS |
| Cox et al. 2018 | To identify patients with unmet PC needs using an PCplanner app | Automatic screening | Yes | 5 | Yes | Literature & ICU team feedback | ICU physician/ Intensivist | Families reported high acceptability (mean CSQ, 14.1) and usability (mean SUS, 21.1) of the PCplanner app. Better Patient-Centeredness of Care Scale (mean increase of 6.6) and the Perceived Stress Scale (mean decrease of 20.8). Earlier SPC referrals mean of 3.9 days) compared to controls (mean of 6.9 days) |
| Mun et al., 2016, 2018 | To identify patients in ICU who may benefit from a timelier SPC | Checklist | No | 6 | Yes | Literature review & ICU clinical expertise & from CAPC | ICU physician/ Intensivist | Earlier identification of, DNR (from 39% to 79%), and goals of care (24% to 70%), & a decrease in ICU (from 5.76 days to 4.92 days) and hospital LOS (17.43 days p to 12.88 days) |
| Henderson et al. 2017 | To analysis the use of the Rotherman Index and LOS as predictors of SPC needs | Checklist | No | 2 | Yes | The Rothman Index-a valid disease diagnostic tool | N/A | The Rotherman Index & LOS identified patients with unmet PC needs. The median satisfaction score was 23 points higher for patients in the intervention group compared to the control group (P < .001) |
| Zalenski et al., 2017 | To identify those at high risk of a complex hospital stay | Checklist | Yes | 7 | No | Previous studies & evidence | ICU physician/ Intensivist | Positive-Of those that screened positive and thus had a PC consultation they were more likely to have a DNR status & more likely to be discharged to a hospice |
| O'Mahony et al., 2017 | To identify those who would benefit from SPC | Checklist | No | 3 | Yes | IPAL-ICU project (CAPC guidelines) & ICU clinicians’ opinions | SPC & ICU Team | Significant cost savings noted- Median ¼ US$6643 vs US$12 399, P < .001. Improvements in some quality-of-care measures, such as increased use of hospice services and formalised advance directives |
| McCarroll, 2018 | To identify those who would benefit from SPC | Checklist | No | 8 | Yes | CAPC guidelines | ICU physician/ Intensivist | Increase in SPC consultation from 10& to 30% |
| Mun et al., 2018 | To identify those who would benefit from SPC | Checklist | No | 6 | Yes | Lit. review & ICU clinical expertise & from CAPC | Automatic | Increased early family meetings, identification of GOC & change of NFR status |
| Ma et al., 2019 | To assess impact of screening | Checklist | No | 9 | No | Previous PC interventions studies | Automatic | NFR/do not intubate occurred earlier & significantly more frequently for those who screened positive |
| Schoenherr et al., 2020 | To identify those who would benefit from SPC | Checklist | No | 11 | Yes | Emerged clinically from assessing patients for PC needs including clinician discussion | ICU physician/ Intensivist | Those referred to SPC had fewer investigations in the last 48 hours of life, more DNR orders, and more deaths outside the ICU in comfort- care settings. |
| Poi et al., 2021 | To measure impact of trigger criteria | Checklist | No | 4 | Yes | Adapted from IPAL-ICU & from CAPC | Automatic | Increased SPC referral rates from 9 to 44 in 2012, from 44 to 47 in 2015.Lower costs per day for those referred to SPC. Continuity of care post-ICU. |
| Sinha et al., 2021 | To identify those who would benefit from SPC | Checklist | No | 4 | Yes | Adapted from IPAL-ICU & from CAPC | ICU physician/ Intensivist | Increased SPC referral rates from 45 to 96 in MICU, from 51 to 77 in SICU. Increased SPC referrals by 113% in MICU & by 51% in SICU compared to the same period the previous year |
| Chung et al., 2022 | To identify those who would benefit from SPC | Checklist | No | 4 | No | Not identified | SPC & ICU Team | Those that had a SPC referral had 8% longer hospital LOS compared to those who received usual care. SPC referrals equalled a significant 21% reduction in total hospital costs compared to those in usual care. |
| Spurry & Alessandrini, 2022 | To identify those who would benefit from SPC | Checklist | No | 19 | Yes | literature (evidence), from IPAL-ICU Report & from CAPC | ICU physician/ Intensivist | Increased SPC referral rates within 24 hrs of ICU admission from 64% to 90% & resulted in earlier access to SPC from 22.39 days to 7.28 days. |
| Helgeson et al., 2023 | To identify those who would benefit from SPC | Checklist | No | 11 | Yes | Literature review, expert opinion & from CAPC | Researchers  All those meeting the criteria were randomised to have SPC referral (intervention) or to the control group | The median satisfaction score was 23 points higher for those in the intervention group compared to the control group (P < .001). ICU stay was 5 days less in the intervention group compared to the control group (95% CI; 1 day to 18 days, P = .018) |
| Davila et al., 2023 | To direct limited resources to highest needs | Checklist | No | 6 | Yes | Not identified | Automatic | 68% of patients had a change in DNR status after SPC consultation. |
| Iguina et al., 2023 | To identify those who would benefit from early PC | Checklist | No | 11 | Yes | Evidence & based on previously reported triggers | SPC & ICU Team | Nearly to half (48.7%) of the ICU patients screened positive for at least one trigger. Patients that met the criteria were more likely to have family meetings within 72 hours, SPC referrals changes to DNR status, palliative extubating, and comfort care measures implemented. |
