# Supplementary material for: Key characteristics of palliative care integration in intensive care units (ICUs): A scoping review
Source: Int J Nurs Stud Adv. 2026 Apr 8;10:100535. doi: 10.1016/j.ijnsa.2026.100535 (PMC13091130; doi:10.1016/j.ijnsa.2026.100535)
Supplement: Supplementary file 2 [file mmc2.doc]

**IJNS & IJNS Advances author checklist** *V2025.1*

| **IJNS Advances AUTHOR CHECKLIST:** You will need to submit a completed version of this checklist plus the checklist from any relevant reporting guideline along with your paper. It is intended to help you to make sure your manuscript meets some basic requirements for the journal. It should be read in conjunction with the guide for authors and is not a replacement for it. Additionally, to help ensure your manuscript is compliant, a [manuscript template](https://relx-elsevier-erms.my.salesforce.com/sfc/p/#1t000000qIy5/a/Tl000000fo5h/5egwGem7CF9fcPQgUICPyp40BQHshDbzsnJjnKjTc8M) is available (also linked from the [guide for authors](https://www.sciencedirect.com/journal/international-journal-of-nursing-studies/publish/guide-for-authors)). | | Insert a tick, page number(s) or give detail |
| --- | --- | --- |
| **Word count** | **The paper is 7000 words or fewer** | ✓  6,107words |
| **Abbreviations** | **My paper does not contain abbreviations.**  Avoid using abbreviations, acronyms or “initialisms” anywhere in the paper other than SI units, common statistical terms. Write out in full at every mention. Abbreviations may be used in tables where they should be defined in a footnote. | ✓  ICU, WHO & European Association of Palliative Care [EAPC], used. |
| **Reporting guideline** | **The paper has been prepared using a recognized reporting guideline appropriate to the method / type of paper. Please identify the reporting guideline used in the box to the right.**  Please consult <https://www.equator-network.org/> to help select an appropriate guide [research and reviews only]. A checklist linked to the reporting guideline should be completed and uploaded as part of your submission. *If there is no applicable guideline, upload a blank file with the words ‘not applicable’ when requested at submission.* | PRISMA-ScR) Checklist see-  Attached |
|  | **My paper does not refer to *reporting* guidelines as a source for *methods.***  Statements such as “This trial was conducted according to the CONSORT guideline” **must not** be included | ✓ |
| **Title** | **The title is in the format ‘Topic / question: design/type of paper’** [not applicable to discussion papers or editorials] | ✓ |
| **Abstract** | **The paper includes a structured abstract of no more than 400 words**  Use headings appropriate to the design of the study. [Not applicable to editorials. Discussion paper abstracts can be unstructured] | ✓ |
|  | **No references are cited in the abstract.** | ✓ |
| **Study registration** | **The abstract includes the study registration including number, the public registry**  **(e.g. ISRCTN), the registration date and date the first participant was recruited.**  If not registered, state ‘not registered’*[[1]](#footnote-2) This should also appear at the end of the methods section [not applicable to narrative reviews and discussion papers, editorials]. | N/A |
|  | **For clinical trials (as** [**defined by the ICMJE**](https://www.icmje.org/about-icmje/faqs/clinical-trials-registration/)**), registration occurred before the first participant was recruited.**  We are unable to consider clinical trials that were registered retrospectively – if uncertain check with editor before submission. | N/A |
| **Key words** | **I have included keywords (maximum 7)**  Use the Medical Subject Headings or Cumulative Index to Nursing and Allied Health terms (see <http://www.nlm.nih.gov/mesh/meshhome.html>). | ✓ |
| **Contribution of the Paper statements** | **I have included up to 6 article “highlights”.**  Under the headings “What is already known” and “What this paper adds” give 2 or 3 (maximum) short, single sentence bullet points (each) summarising key contributions. No references are to be cited. | ✓ |
| **Multiple publications** | **I have explicitly mentioned other publications from the same study in my paper**  Other published and in press accounts of the study from which data in this paper originate are referred to in the paper (author details can be redacted for review if desired) and the relationship between this and other publications from the same study is made clear in the paper. [see below] | N/A |
| **Ethical approval and informed consent** | **I have given details of the ethical approval, including the body that granted it and reference number at the end of my methods section.**  This should include confirmation of informed consent by participants and / or elaboration of the basis for any exception. [**r**esearch papers only, not applicable for reviews and discussion papers]. | N/A |
| **Statistical reporting** | **I have followed journal guidance on reporting statistics, confidence intervals and statistical significance (summarized below)** | ✓ |
|  | - Confidence intervals can be used as the basis for inference without reference to statistical significance & ‘p-values’ - Where p values are reported, measures of effect or association and confidence interval are reported alongside all significance tests / p values (including in the abstract). - Exact p-values are stated to an appropriate degree of precision (typically no more than 3 decimal points). - The term ‘statistically significant’ (not just ‘significant’) is used to refer to the result of tests. - p-values>0.05 (not statistically significant) are not interpreted as demonstrating no effect/association.   Please consult   - Basic statistical reporting for articles published in Biomedical Journals <https://doi.org/10.1016/j.ijnurstu.2014.09.006> for general guidance on statistical reporting - Statistical significance testing and p-values A discussion paper and position statement. <https://doi.org/10.1016/j.ijnurstu.2019.07.001> for our policies on reporting point estimates, confidence intervals and p-values / significance tests |  |
| **Qualitative findings** | **Key quotations to support inferences are given with give meaningful (anonymous) individual subject identifiers for each quotation used.** | N/A |
| **Use of generative Artificial Intelligence** | **I have reviewed the Elsevier** [**Generative AI policy**](https://www.elsevier.com/about/policies-and-standards/generative-ai-policies-for-journals) **and, if relevant, I have included a “Declaration of generative AI and AI-assisted technologies in the manuscript preparation process” before the reference list.** | ✓ |
| **Please provide below references for any other publications based on data from the same study that you report here. Describe the relationship to the current study (see also ‘multiple publications’, above).**  e.g. “A previous publication from this study showed that carrots were effective in preventing blindness. This study reports on a range of other health benefits, which were not reported in our earlier paper [give full reference / doi link].”  *To assist editors, upload copies of papers where the abstract / full text is not readily available (including those under review elsewhere, which will be treated in strict confidence).*  *Where your paper is based on analysis of a publically available data set or is part of a series of publications from a large cohort study (or similar) you can be selective in the references you provide and give a more general account of how this paper relates to others but it is essential that editors are able to verify the unique contribution of the paper you are submitting.*  *If unsure about declarations we encourage you to err on the side of openness and suggest you consult Norman, I., Griffiths, P., 2008. Duplicate publication and 'salami slicing': Ethical issues and practical solutions. International Journal of Nursing Studies 45 (9), 1257-1260.* | | |
| Insert details of previous publications *from this study* below.  N/A | | |

*© Peter Griffiths, Ian Norman – 2025 (version September 2025)*

*This work is licensed under a*[Creative Commons Attribution 4.0 International License.](http://creativecommons.org/licenses/by/4.0/)

1. While the journal endeavours to maintain a double blind-review process as far as possible, we give priority to transparent reporting and prospective registration. As it is important that reviewers can verify that reporting is complete and consistent with protocols to avoid (for example) selective outcome reporting or undocumented protocol changes, authors are not permitted to redact registration numbers for review. By choosing to submit you acknowledge this exception to anonymity in the review process. [↑](#footnote-ref-2)
