# Supplementary material for: Key characteristics of palliative care integration in intensive care units (ICUs): A scoping review
Source: Int J Nurs Stud Adv. 2026 Apr 8;10:100535. doi: 10.1016/j.ijnsa.2026.100535 (PMC13091130; doi:10.1016/j.ijnsa.2026.100535)
Supplement: Supplementary file 4 [file mmc4.docx]

**SUPPLEMENT FILE S1**

**Search String and Index Terms**

|  | ***Concept*** | ***Index Term: CINAHL*** | ***Index Term: Medline*** | ***Index Term: PsycINFO*** | ***Index Term: EMBASE*** | ***Index Term: Cochrane*** |
| --- | --- | --- | --- | --- | --- | --- |
| #1 | PC | (MM "PC") OR (MM "Hospice and Palliative Nursing") | (MH "PC") OR (MH "Hospice and PC Nursing") OR (MH "Palliative Medicine")  OR (MH "Terminal Care") | DE "PC" | palliative AND care AND in AND proximity AND to AND integration | MeSH descriptor:  [Palliative Medicine] |
| #2 | ICU | (MM "Intensive Care Units+")  (major concept & explode) | (MH "Intensive Care Units+") OR ("Critical Care Nursing") OR (MH "Critical Care") | DE critical care | ('intensive care'/exp OR 'intensive care unit'/exp) AND – AND this AND is AND the AND location | MeSH descriptor: [Critical Care] |

**Index terms**

| **Database – Embase** | |
| --- | --- |
| **Concept 1= PC**  **INDEX Terms** =  palliative AND care AND in AND proximity AND to AND integration  **Keywords** = (palliativ* OR hospice* OR terminal* OR 'end of life*') NEAR/3  (integrat* OR merge* OR unifi* OR combine*) | **Concept 2: ICU**  INDEX Terms= 'intensive care unit'/exp OR 'intensive care'/exp – this is the location  Keyword= ‘critical care*’ OR ‘intensive care*’ OR ‘intensive therapy*’  OR ICU OR ICUs OR CCU OR CCUs OR ‘acute care*’ |
| **Database – CINAHL** | |
| **Concept 1= PC**  **INDEX Terms** =  (MM "PC") OR (MM "Hospice and Palliative Nursing")  **Keyword** = palliat* OR terminal OR Critically ill OR VI end of life* OR end-of-life*  OR life-sustain* OR life sustain* OR hospice* | **Concept 2: ICU**  **INDEX Terms** = (MM “Intensive Care Units+”) (major concept & explode)  **Keyword** ="Critical Care*" OR "Intensive Care*" OR "Acute care*"  OR ICU OR ICUs OR CCUs OR CCU |
| **Database – MEDLINE** | |
| **Concept 1: PC**  **INDEX Terms =**  (MH "PC") OR (MH "Hospice and PC Nursing")  OR (MH "Palliative Medicine")  OR (MH "Terminal Care")  **Keywords =** palliat* OR terminal OR Critically ill OR "end of life* OR end-of-life*  OR life-sustain* OR life sustain* OR hospice* OR emotional support OR mortal support | **Concept 2: Intensive Care**  INDEX Terms= MM "Intensive Care Units+" (explode and major concept)  OR ("Critical Care Nursing") OR (MH "Critical Care")  Keywords = "Critical Care*" OR "Intensive Care*" OR "Acute care*"  OR ICU OR ICUs OR CCUs OR CCU |
| **Database – PsycINFO** | |
| **Concept 1= PC**  INDEX Terms=  DE PC  Keywords in AB (abstract)= palliat* OR terminal OR Critically ill OR VI end of life*  OR end-of-life* OR life-sustain* OR life sustain* OR hospice* OR emotional support  OR mortal support | **Concept 2: ICU**  INDEX Terms= DE critical care  Keyword AB (abstract s= "Critical Care*" OR "Intensive Care*"  OR "Acute care*" OR ICU OR ICUs OR CCUs OR CCU |
