# Supplementary material for: Key characteristics of palliative care integration in intensive care units (ICUs): A scoping review
Source: Int J Nurs Stud Adv. 2026 Apr 8;10:100535. doi: 10.1016/j.ijnsa.2026.100535 (PMC13091130; doi:10.1016/j.ijnsa.2026.100535)
Supplement: Supplementary file 5 [file mmc5.docx]

**Supplement file S2: Thematic Analysis**

**Theme Screening Criteria**

| **Higher SubThemes** | **Subthemes** | **Codes** | **References** |
| --- | --- | --- | --- |
| **Varied Screening responsibility/Role** | SPC screened using the tool | - Embedded palliative care team manually reviewed MICU patient’s HER - Screening conducted by embedded palliative care nurse practitioner - A Palliative Care ANP | O'Mahony et al., 2017 |
|  | ICU Staff screened using the tool | - Screening by ICU Nurse-in-Charge or designated staff. - SICU staff participating voluntarily. - Daily checklist incorporated into ICU rounds. - Nursing staff responsible for administering and recording screening. - Bedside nurses performing the screening. - ICU team conducting daily rounds to reassess criteria. - Later transitioned to the ICU Nurse Practitioner and ICU Nurse-in-charge to ensure consistency | Anderson et al., 2017; Jenko et al., 2015; Kyeremanteng et al., 2020; Mun et al., 2016, 2018; Paré et al., 2021; Poi et al., 2021; Martz et al., 2020; Zalenski et al., 2017 |
|  | Screening-MDT / Joint rounding using the tool | - Screening during MDT rounds - Risk factors assessed jointly: Implementation of the PNST during MDT rounds - Screening conducted by PC team during NS-ICU rounds | Creutzfeldt et al., 2016; Davila et al., 2023; Helgeson et al., 2023; Iguina et al., 2023; Liu et al., 2022; McCarroll, 2018; Poi et al., 2022 |
|  | Automatic screening of the tool | - EHR-based e-triggers (e.g., PCplanner) for automatic identification - Auto-consultation for COVID-19 ECMO patients | Cox et al., 2018; Poi et al., 2022 |
|  | Researcher-led screened using the tool | - Screening conducted by study authors. - EMR review by research team members. - Research coordinators reviewing ICU records using defined criteria. - Project leader chart review based on screening criteria. | Braus et al., 2016; Carson et al., 2016; Constantine et al., 2016; Ma et al., 2019 |
|  | Unknown | - Study did not explicitly state who did the screening | Chung et al., 2022; Henderson et al., 2017 |
| **Systematising Early Identification of Palliative Needs in ICU using a Screening Tools & Triggers** | Systematic screening tools | - **Predefined triggers** (e.g., IPAL-ICU recommendations, COVID-19–specific criteria). - **Prognostic / frailty scales** (SOFA, APACHE II, ProVent 14, PPSv2, Palliative Risk Score). - **ICU-specific tools** (PNST, NEST, 11-item trigger tools, 9-item tools). - **EMR integration**: electronic triggers and checklists for daily or weekday screening. - **Referral pathways**: Positive screens leading to SPC referral or proactive family meetings. - **Timing**: Screening performed within 12 hours of admission, within 24 hours, daily, or at specified ICU days. - **Referral process:** A standardised screening and referral process was implemented | Anderson et al., 2017; Braus et al., 2016; Carson et al., 2016; Chung et al., 2022, 2021; Constantine et al., 2016; Cox et al., 2018; Creutzfeldt et al., 2015; Davila et al., 2023; Helgeson et al., 2023; Henderson et al., 2017; Iguina et al., 2023; Jenko et al., 2015; Kyeremanteng et al., 2020; Liu et al., 2022; Ma et al., 2019; Martz et al., 2020; McCarroll, 2018; Mun et al., 2018; O'Mahony et al., 2017; Poi et al., 2022; Roczen et al., 2016; Schoenherr et al., 2020; Zalenski et al., 2017 |
|  | Clinician-led interventions | - Screening results guide **goals-of-care discussions**. - Early **symptom management** based on identified needs. - Initiation of **proactive family meetings**. | Anderson et al., 2017; Rivet et al., 2018 |
|  | Mortality / risk-based screening | - Patient factors: age, comorbidities, disease severity. - Prognostic scoring systems: SOFA, APACHE II. - Condition-based identification: diseases with poor prognosis. - Mortality/risk tools: Palliative Risk Score, Rothman Index (RI), ProVent 14 score. | Carson et al., 2016; Cox et al., 2018; Davila et al., 2023; Gabda et al., 2021; Helgeson et al., 2022; Henderson et al., 2017; Iguina et al., 2023; Ma et al., 2019; Mun et al., 2016 |
|  | Transitional care triggers | - Dialysis withdrawal; hospice eligibility; advance care planning | Anderson et al., 2017; Poi et al., 2022 |
|  | Holistic / psychosocial triggers | - Family/caregiver burden, psychological distress, surrogate absence | Cox et al., 2018; Gupta et al., 2022 |
|  | Ethical triggers | - Autonomy, dignity, beneficence, treatment futility | Adler et al., 2017; Poi et al., 2022 |
| **Workflow Integration of Palliative Care Screening** | Screening during rounding by MDT | - Repeated weekday screening: medical team informed; researchers notified PC clinician each morning. - PNST incorporated into daily interdisciplinary rounds - Four screening questions read aloud, answered by any team member, and addressed/documented. - Screening criteria integrated during MDR (Multidisciplinary Rounds); initial screening within 12 hours of admission; positive screens randomised to early SPC or standard care. - Joint screening for COVID-19 admissions by ICU and PC teams. | Braus et al., 2016; Carson et al., 2016; Creutzfeldt et al., 2015; Davila et al., 2023; Iguina et al., 2023; Helgeson et al., 2022; Liu et al., 2022: O'Mahony et al., 2017; Metaxa et al., 2021 |
|  | Nursing-Driven Screening Integration | - Nurse-led bedside screening during rounds triggers ICU interventions or SPC referrals. - Embedded into daily nursing routines; nurse champion manages e-trigger alerts, coordinates with physicians, and documents referrals. - PPSv2 documentation daily; verbal reporting during interdisciplinary rounds. - Proactive case-finding by PC advanced practice nurses during rounds. | Anderson et al., 2017; Cox et al., 2018; Jenko et al., 2015; Martz et al., 2020; McCarroll, 2018 |
|  | Positive Screens Trigger Automatic Specialist Referral | - Positive screened resulted in SPC, pathway positive screen equalled specialist referral | Chung et al., 2022; Kyeremanteng et al., 2020; Poi et al., 2022 |
|  | Digital Workflow Integration | - EMR-based alerts for patients meeting PC criteria, prompting review/referral. - Automated electronic risk scoring (Rothman Index) triggers chart review. - Screening embedded into EHR; nurses completed tool daily on weekdays | Constantine et al., 2016; Cox et al., 2018; Henderson et al., 2017 |
|  | Physician Oversight | - ICU attending reviews identified triggers and decides on SPC consultation. - Specialist referrals only initiated if approved by attending or primary team. - Specialist palliative care consultation initiated at the discretion of MICU clinicians - Consultee services were used at the intensivist’s discretion - Final decision for specialist referral made by attending physicians - Critical Medicine physicians made decisions on referrals - Specialist referrals were only initiated if approved by the primary team. - Screening and patient identified during rounding | Anderson et al., 2017; Braus et al., 2016; Carson et al., 2016; Chung et al., 2022; Constantine et al., 2016; Cox et al., 2018; Creutzfeldt et al., 2015; Davila et al., 2023; Helgeson et al., 2023; Iguina et al., 2023; Liu et al., 2022; Ma et al., 2019; Martz et al., 2020; McCarroll, 2018; O'Mahony et al., 2017; Schoenherr et al., 2020; Zalenski et al., 2017 |
|  | Administrative | - Unit secretary places screening tool in bedside book daily. | McCarroll, 2018 |
|  | Limited Integration into Practice | - Screening was conducted retrospectively rather than integrated into practice | Constantine et al., 2016 |
| Needs Assessment Strategies | Screening questions or scales | Screening questions/scales:   - “Would you be surprised if this patient died during this hospital stay?” - “Are you worried the patient will die during this hospitalisation?” - Use of the NEST tool (Needs, Existential concerns, Symptoms, Therapeutic interactions) to identify palliative care needs as perceived by family members | Creutzfeldt et al., 2015; Crooms & Gelfman, 2020; Cox et al., 2018, 2021; Mun et al., 2016; Sinha et al., 2021; Schoenherr et al., 2020 |
| **Reliability, Validation & feasibility** |  | - Derived from IPAL-ICU/CAPC frameworks and expert review. - Usability assessed and informed by prior studies using nationally recognised prognostic screening tools. - E-triggers designed/validated during development for clinical relevance and outcome association (disability, readmission, symptoms, mortality). - Pilot-tested with ICU physicians and neuro-ICU nurses via one-on-one meetings, electronic communication, and work-in-progress presentations. - PPSv2 recognized as reliable, valid, and simple to use. - Clinician surveys highlighted “criteria-triggered consults” as feasible. - Evidence-based triggers consistently prioritised across studies. - Limited systematic validation of screening accuracy; quality assurance performed only on subsets of data. - Selected for their clinical relevance, reliability of temporal detection, and established associations with patient outcomes (disability, readmission, symptoms, mortality) | Braus et al., 2016; Constantine et al., 2016; Cox et al., 2018; Creutzfeldt et al., 2015; Crooms & Gelfman, 2020; Henderson et al., 2017; Jenko et al., 2015; Kyeremanteng et al., 2020; Ma et al., 2019; Martz et al., 2020; McCarroll, 2018: Zalenski et al., 2017 |
| **Implementation Challenges** | Barriers | - Heterogeneity in who screens - Unclear workflows - Time pressures - Competing priorities - SPC stigma, resistance to referrals; limited organisational support; Limited adoption; SPC stigma; physician biases; clinician disagreement with referral criteria; uncertainty about which triggers to apply | McCarroll, 2018; Cox et al., 2018; Iguina et al., 2023; Wiencek, 2024; Zalenski et al., 2017 |

*Specialist palliative Care (SPC)

**Table: Trigger Types**

|  | COVID- 19 | From or to  long - term  care | At home ADL dependencies,  poor functional status or  Pre-existing tracheostomy | Progressive neurological disease or  severe cognitive impairment | Advanced  /metastatic cancer or new cancer | Cardiac,  respiratory arrest with neuro issues | Multiple organ system failure APACHE  Escalate needs | End- stage organ  disease | Acute shock or Severe  sepsis  ≥ 12  − 24 ℎ𝑟𝑠 | Acute respiratory failure, on prolonged ventilation  Intra cranial bleed,  severe trauma | Hospital  /ICU stay  ≥ 5 -14 days or multi-  re-admissions | Aged >70-80 with  1-2 or > comorbidities  or poor prognosis  /complex case | Conflict,  family request,  perceived futility, DNR. need for social support. refractory symptoms |
| --- | --- | --- | --- | --- | --- | --- | --- | --- | --- | --- | --- | --- | --- |
| Creutzfeldt et al., 2015 |  |  |  | ***** | ***** | ***** |  |  |  |  | ***** | ***** |  |
| Jenko et al., 2015 |  |  | ***** |  |  |  |  |  |  |  |  |  |  |
| Carson et al., 2016 |  |  |  |  |  |  |  |  |  | ***** |  | ***** |  |
| Constantine et al., 2016 |  |  |  |  | ***** | ***** | ***** |  |  |  | ***** |  |  |
| Braus et al., 2016 |  | ***** |  | ***** | ***** | ***** |  | ***** |  |  | ***** | ***** |  |
| Anderson et al., 2017 |  |  |  | ***** | ***** |  | ***** | ***** |  | ***** |  |  |  |
| Henderson et al., 2017 |  |  |  |  |  |  |  | ***** |  |  | ***** |  |  |
| O'Mahony et al., 2017 |  |  |  |  | ***** |  |  | ***** |  |  | ***** |  |  |
| Zalenski et al., 2017 |  | ***** | ***** | ***** | ***** | ***** |  |  |  |  | ***** | ***** |  |
| Cox et al., 2018 |  |  | ***** | ***** |  | ***** | ***** |  | ***** |  | ***** | ***** |  |
| McCarroll, 2018 |  |  |  | ***** | ***** |  | ***** | ***** |  |  | ***** | ***** | ***** |
| Mun et al., 2018 |  |  |  | ***** | ***** |  | ***** |  |  |  | ***** |  | ***** |
| Ma et al., 2019 |  | ***** | ***** | ***** | ***** | ***** | ***** | ***** | ***** | ***** | ***** |  |  |
| Martz et al., 2020 |  | ***** | ***** | ***** | ***** | ***** |  | ***** |  | ***** | ***** | ***** | ***** |
| Schoenherr et al.,2020 | ***** |  |  |  |  |  | ***** |  |  |  |  | ***** | ***** |
| Chung et al., 2022 |  |  | ***** |  | ***** |  | ***** | ***** |  |  |  |  |  |
| Liu et al., 2022 |  | ***** |  | ***** | ***** |  |  | ***** |  |  |  | ***** |  |
| Poi et al., 2022 |  |  |  | ***** |  |  | ***** | ***** |  |  |  |  |  |
| Davila et al., 2023 |  |  |  | ***** | ***** |  | ***** |  |  | ***** |  | ***** | ***** |
| Helgeson et al., 2023 |  |  | ***** | ***** | ***** | ***** | ***** | ***** |  |  |  | ***** |  |
| Iguina et al., 2023 |  |  |  | ***** | ***** | ***** | ***** | ***** |  | ***** | ***** | ***** | ***** |

**Theme: Embedded Practices**

| **Higher Sub Themes** | **Subthemes** | **Codes** | **References** |
| --- | --- | --- | --- |
| **Core palliative care practices** | Symptom & Pain assessment and management; early goals-of-care discussions; early goals-of-care support; advance directive documentation; Concordant Care; Values-based planning; surrogate decision-making, Advanced Care Planning Note, identifying uncontrolled symptoms | - Documenting patients’ wishes - Having family meetings within 72 hours of ICU admission and assistance to families in transitioning goals of care - Enhancing pain assessment at ICU admission and the response to therapy at 48 hr - Focused on the eight domains of palliative care as per Clinical Practice Guidelines for Quality PC – Structure and Process of Care; Physical Aspects of Care; Psychological and Psychiatric Aspects of Care; Social Aspects of Care - Advance care planning conversation involved assessing the decision-making capacity of the patient - Advance care planning note - Common PC tasks completed included information giving, coping support, and goals of care discussions - Completing an advance directive and addressing code status. - Assist in determining goals of care, such as transitioning patients to home care, hospice, or skilled nursing facilities. - Aimed to facilitate early goals-of-care discussions to address patient preferences and reduce suffering - Discuss patient care plans, symptom management, and family expectations - The program was designed to provide linguistic and culturally concordant care, in response to the first COVID-19 - Daily review of palliative care needs | Anderson et al., 2017; Baker et al., 2015; Braus et al., 2016; Carson et al., 2016; Chung et al., 2022; Cralley et al., 2022; Davila et al., 2023; Frontera et al. 2015; Gupta et al., 2022; Helgeson et al., 2023; Iguina et al., 2023; Liu et al., 2022; Mehta et al., 2023; Mun et al, 2017; Paré et al., 2021; Poi et al., 2022; Roa et al., 2023; Soper et al., 2023; Vig, 2019; Vuong et al., 2019 |
|  | Time sequenced processes | - C&CB, day 1-3 GOC conversations - Advanced directive documentation - Proactive family meetings - Social support if positive screened for PC needs | Constantine et al., 2016; Paré et al., 2021; Metaxa et al., Mun et al., 2016, 2017; Vuong et al., 2019 |
|  | Family Engagement & Communication | - Early family meetings - Goal-concordant conversations - Family support, unified messaging - ICUconnect app for family to submit PC needs - identifying family distress - Supporting decision making | Anderson et al**.**, 2017**; Adler et al., 2017; Carson et al., 2016;** Cox et al.**,** 2021**;** Iguina et al., 2023; Mun et al., 2017;  **Hernandez-Zambrano et al., 2024** |
|  | Joint Decision-Making & Communication | - Shared decision-making - Communication tools | Braus et al., 2016; Gupta et al., 2022 |
|  | Existential support | - Spiritual support& care - Spiritual, Religious, and Existential Aspects of Care | Anderson et al**.**, 2017**; Davila** et al., 2023; **Dolmans et al., 2023**; Helgeson et al., 202; 3**Mun et al., 2016;** |
|  | Embedded Role & champions | - Physicians; ANP /specialist nurses - Social worker - chaplains’ collaboration - Dedicated Spanish speaking Palliative Care person, - Champions | Anderson et al., 2017; Baker et al., 2015; Braus et al., 2016; Cralley et al., 2022; Davila et al., 2023; Gabda et al., 2021; Jenko et al., 2015; Martz et al., 2020; McCarroll, 2018; Mehta et al., 2023; O'Mahony et al., 2017; Poi et al., 2022; Schockett et al., 2021; Soper et al., 2023; Vuong et al., 2019 |
|  | Continuity of Care | - Post-ICU follow-up - Terminal discharge planning | Cox et al., 2018; Kahveci, 2017 |
|  | Stakeholder engagement | - Collaboration with ICU leadership - Multiple meetings with key stakeholders - Active engagement with stakeholders - SPC hired to ensure services | Anderson et al., 2017; Constantine et al., 2016; Cox et al., 2018, 2021; Cralley et al., 2022; Creutzfeldt et al., 2015; Davila et al., 2023; Grouls et al., 2022**;** Henderson et al., 2017; Jenko et al., 2015; Iguina et al., 2023; Kyeremanteng et al., 2020: Martz et al., 2020; McCarroll, 2018; Mehta et al., 2023; Mun et al., 2016, 2017; O'Mahony et al., 2017; Paré et al., 2021; Poi et al., 2022; Schockett et al., 2021; Schoenherr et al., 2020; Sinha et al., 2021; Soper et al., 2023; Vig, 2019; Vuong et al., 2019: Zalenski et al., 2017 |
|  | Implementation Challenges | - Workflow complexity - Curative culture - Misconceptions - Varied findings - SPC limited resources - Workflow alignment - Modifying SPC service schedules - Requires a considerate - Stepwise approach to overcome barriers | Baker et al., 2015; Davila et al., 2023; Iguina et al., 2023; Roa et al., 2023; Soper et al., 2023; **Poi et al., 2022; Gupta et al., 2022** |
| **Workflow Integration** | Core palliative care practices | - ICU teams systematically assess symptoms - Initiate goals-of-care discussions, - Document advance directives - Engage families to align care with patient values - Family Engagement & Communication - Early family meetings - Goal-concordant conversations - Family support | Carson et al., 2016; Frontera et al., 2015; Mehta et al., 2023; Roa et al., 2023; Soper et al., 2023 |
|  | Time sequenced processes | - Use of Care & Communication Bundle (C&CB) - Applied across ICU days 1, 3, 5 to integrate tasks such as pain assessment, decision-maker identification, and family support - C&CB improved documentation but relational aspects inconsistent - C&CB, day 1-3 GOC conversations, advanced directive documentation, proactive family meetings & social support if positive screened for PC needs | Constantine et al., 2016; Mun et al., 2016, 2017; Vuong et al., 2019 |
|  | Bridging Roles | - Palliative care nurse practitioner was embedded on the - neuroscience ICU team - ICU nurse champions identify unmet needs, facilitate communication, and connect specialist care with bedside practice - Nurse champions, palliative care liaisons | Davila et al., 2023; Mehta et al., 2023; Poi et al., 2022; Soper et al., 2023 |
| **Stakeholder Engagement** | Leadership involvement | - Engaging ICU leadership and frontline staff to adapt palliative practices to local context; | Anderson et al., 2017; Cralley et al., 2022; Iguina et al., 2023; Gupta et al., 2022 |
| **Challenges** | Systemic barriers | - Adoption varied across units - Limited policy support and resource constraints hindered full integration - Workflow complexity - Curative culture, - Misconceptions, - Varied findings, - Workflow alignment |  |
| **Relational Competence** | Ethical alignment, communication, role modelling | Integration depends on visible role models, interprofessional communication, and ethical alignment alongside protocols; ensures patient values are respected | Baker et al., 2015; Roczen et al., 2016; Frontera et al., 2015; O’Connell & Maier, 2016 |
| **Education** | Continuous training, skill reinforcement | Ongoing education supports clinician confidence, ethical decision-making, communication, and real-time application of palliative principles; central to maintaining alignment with core ICU values | Jenko et al., 2015; Mehta et al., 2023 |

**Theme Education**

| **Suthemes** | **Codes** | **References** |
| --- | --- | --- |
| **Education Resources** for ICU staff | - Centre to Advanced Palliative Care (CAPC) module - Structured communication program - Online courses:   - VitalTalk; Education in Palliative and End-of-Life Care Program – ICU (EPEC), & the End-of-Life Nursing Education Consortium (ELNEC); Critical Care Communication skills program (“C-3”); the IMPACT-ICU program implemented communication workshop | Anderson et al., 2017; Baker et al., 2015; Crooms & Gelfman, 2020; Gupta et al., 2022; Mehta et al., 2023; Soper et al., 2023 |
| **Practical Application** | - Focused on how to use the PPSv - ICU clinician training on   - Testing to develop familiarisation with Palliative Care Needs Screening Tool (PNST); identifying appropriate patients with unmet PC needs; multifaceted educational intervention implemented to promote the adoption of the Care and Communication Bundle | Constantine et al., 2016; Cox et al., 2018; Creutzfeldt et al., 2015; Jenko et al., 2015; Soper et al., 2023 |
| **Diverse Teaching Formats** | - Online modules - Bedside coaching staff - Case-based teaching; Workshops - Use of champions/mentors - Instructional videos - Role modelling and supervised practice - Lectures - PowerPoint - Stimulation - Webinars - Posters - Instructional videos to enhance family communication skills - Academic detailing; 15-minute segments - Described diverse teaching strategies in various interventional studies - Expert support/tuition - Monthly sessions to review C&CB - Use of biweekly ‘candy reminders’ as positive reinforcement to sustain engagement - Train-the-Trainer Program for Nurse Leaders. | Anderson et al., 2017; Aslakson et al., 2017; Cralley et al., 2022; Cox et al., 2018, 2021; Hernandez-Zambrano et al., 2024; Iguina et al., 2023; Jenko et al., 2015; Kyeremanteng et al., 2018; McCarroll, 2018; Metaxa et al., 2021; Mun et al., 2016, 2017, 2018**;** O'Mahony et al., 2017; Poi et al., 2022; Sady et al., 2021; Schockett et al., 2021; Soper et al., 2023; Vig, 2018; Vuong et al., 2019 |
| C**ommunication Skills & Training** | - GOC/EOL workshops - Communication workshops - Developing skills for family conversations about prognosis and treatment decision - Enhancing staff competence in family meeting engagement - Training intensivists in PC communication techniques - Teaching frameworks for opening conversations - “Ask-Tell-Ask”, to respond to emotions using the NURSE (Naming, Understand, Respect, Support, Explore) - Use of the Psychosocial Assessment and Communication Evaluation (PACE) to improve communication skills - Communication facilitators to enhance ICU staff skills - Use of the ‘Best Case/Worst Case’ tool to guide family communication - Need for upskilling in PC communication including senior ICU staff - Training in delivering bad news, discussing end-of-life care, conveying compassion and making decisions about limiting life support - Training in communication for time-limited trials and surrogate decision-making - Critical Care Communication Skills program (C-3) to train ICU clinicians in communication | Anderson et al., 2017; Aslakson et al., 2017; Carson et al., 2016; Curtis et al., 2022; Creutzfeldt et al., 2015; Crooms & Gelfman, 2020; Dolmans et al., 2023; Gupta et al., 2022; Hernandez-Zambran et al., 2024; Kahveci, 2017; ; Kyeremanteng et al., 2018, 2020**;** Metaxa et al., 2021; Mun et al., 2017; Poi et al., 2022; Roczen et al., 2016; ; Sady et al., 2021; Schockett et al., 2021; |
| **Core Content and Skills in Palliative Care** | - Training in family support - Education on early SPC integration - Learning to conduct Goals-of-Care (GOC) discussion - Understanding EOL ethical principles - Addressing conflicts - Developing shared decision-making & communication skills - Addressing misconceptions - Education on PC's role in ICU - Developing practical skills in PC symptom assessment & management - Bereavement support & shared decision making - Education to enhance core primary & practical PC skills - Need for PC skill development - Training intensivists and neurosurgery residents in primary palliative care skills - Bedside nursing education for symptom assessment - Reviewing C&CB to reinforce core PC principle - Partnered with the Spanish Language Care Group to train clinicians on communication strategies and serious illness conversations - Spiritual aspects | Anderson et al., 2017; Aslakson et al., 2017**; Braus et al., 2016**; Curtis et al., 2022; Cralley et al., 2022; **Creutzfeldt et al., 2015;** Davila et al., 2023; Frontera et al., 2015; Hernandez-Zambrano et al., 2024 Iguina et al., 2023; Jenko et al., 2015; Kahveci, 2017; Kim et al., 2022; Kyeremanteng et al., 2018, 2020; Mercadante et al., 2018**;** Mehta et al., 203; Metaxa et al., 2021; Mun et al., 2016**,** 2017**;** O’Connell & Maier, 2016; Poi et al., 2022; Rivet et al., 2018; Roczen et al., 2016; Sady et al., 2021; Schockett et al., 2021; Soper et al., 2024; Vuong et al.**,** 2019**;** Wiencek, 2024 |
| **Burnout & Moral Distress** – | - Stress mitigation - Addressing ICU clinicians’ emotional challenges - Education targeting emotional and moral stressors for ICU clinician | Baker et al., 2015; Mehta et al., 2023; Mercadante et al., 2018 |
| **Family Education** | - Provision of informational materials for families - Communication of PC principles - Using VALUE format for family education/interactions - Encouraging families to watch “Goals-of-Care” video - Education for patients/families on prognosis and treatment benefits, on risks & misconceptions of PC - Need for family education - Educating families about the patient’s condition, treatment options, and prognosis to facilitate shared decision-making - Coaching to empower families in decision-making | Aslakson et al., 2017; Carson et al., 2016; Cox et al., 2018, 2021; Crooms & Gelfman, 2020; Frontera et al., 2015; Gupta et al., 2022; Krishnappa et al., 2018; Mun et al., 2017, 2018; Paré et al., 2021; Sady et al., 2021 |
|  |  |  |
| **Targeted Education for Specific ICUs** | - ICU-specific (trauma, neuro, cardiac) - Targeted primary PC training for neurologists and neuro ICU clinicians - Need for ICU MD and Ph.D. programs in SPC | Gupta et al., 2022; Mun et al., 2016; Sady et al., 2021; O’Connell & Maier, 2016 |
| **Nurse-Led Education** | - Bedside nurse led education - Champions raise PC awareness - Nurse leaders were trained to coach bedside nurses in identifying and addressing palliative care needs | Anderson et al., 2017; Crooms & Gelfman, 2020 |
| **Ethical and Relational aspects of Care Navigation** | - Ethics consultation - Moral decision-making - Addressing disparities in care - Ethical Training eg., on ethical and legal aspects of limiting life-sustaining treatment - Need for ethical training; ethical aspects of withdrawing life-sustaining treatments - Addressing cultural barriers and misconceptions to reduce PC stigma - Ppromoting equitable access or addressing societal misconceptions - Ethics-focused debriefing sessions to support clinicians’ moral reflection and coping | Anderson et al., 2017; Aslakson et al., 2017; Curtis et al., 2022; Gupta et al., 2022; Hernandez-Zambrani et al., 2024; Mercadante et al., 2018; Roczen et al., 2016; Sady et al., 2021; Soper et al., 2023; Vig, 2918. |
| **Enhance Acceptability and Engagement with PC** | - Reducing stigma surrounding PC, staff report they need more training after feedback - Teaching ICU staff about need for PC - Need for surgeon education/training to enhance SPC acceptability and engagement - Addressing barriers (unrealistic expectations, care disagreements) to enhance SPC integration and engagement - Need or recommendations for ICU staff PC training - Need for educational interventions because of knowledge or communication deficits - Need for PC skill development - Strategies to overcome cultural barriers and misconceptions to reduce PC stigma - Training SPC clinicians in ICU medicine and culture to enhance collaboration and integration - Reframe PC as quality-of-life - Address cultural barriers to increase acceptability & cooperation of its integration - Need for targeted interventions to improve engagement - Education at all levels to improve collaboration, integration, and acceptability of PC in ICU - Increasing public awareness and organising outreach program - Coaching family members to ask questions and promote engagement in care - Educational presentation dispelling myths - Reviewing policy, and promoting awareness of palliative care benefits in the ICU | Braus et al., 2016**;** Adler et al., 2017; Aslakson et al., 2017; Baker et al., 2015; Carson et al., 2016; Cox et al., 2021; Curtis et al., 2022; Crooms & Gelfman, 2020; Gupta et al., 2022; Kim et al., 2022; Kyeremanteng et al., 2018; Mercadante et al., 2018; McCarroll, 2018; Metaxa et al., 2021; Mun et al., 2016; O’Connell & Maier, 2016; Rivet et al., 2018; Sady et al., 2021; Mercadante et al., 2018; Zalenski et al., 2017 |
| **Challenges** | - Resistance to training due to time constraints, cost, home responsibilities, staffing and scheduling issues - Organisational resistance - Time Constraints - ICU staff often face competing demands - Misunderstandings equating palliative care with end-of-life care or hospice led to resistance among ICU staff - Misconceptions about PC’s role delayed staff engagement with educational initiatives and hindered the adoption of PC practices. - Resistance from ICU leadership or staff to adopt palliative care education due to perceived conflicts with the ICU’s curative focus - skepticism about the value of palliative care, limited the initial uptake of education programs - Limited availability of trained educators, funding, and materials for palliative care education. - Absence of national-level frameworks and resources for palliative care education - Differences in staff motivation and willingness to participate in education programs. - ICU clinicians often prioritise acute care tasks over PC education - Inconsistent approaches to PC education across institutions led to variability in implementation and outcomes. - Financial constraints and limited staffing made it difficult to implement comprehensive education programs | Anderson et al., 2017; Baker et al., 2015; Constantine et al., 2016; Gupta et al., 2022; Jenko et al., 2015; McCarroll, 2018; Metaxa et al., 2021 |

**Theme Increased Specialist Palliative Care (SPC) Collaboration**

| **Subthemes** | **Codes** | **References** |
| --- | --- | --- |
| Joint ICU–PC Rounding | - Daily/weekly rounds - Proactive collaboration rounds - Co-management remotely via teleconference - Physician with expertise in ethics, palliative care, and geriatrics attending morning round - Weekly Interprofessional meetings were held between the palliative care team, intensivists, neurosurgeons, ICU nurses, and medical social workers | Kyeremanteng et al., 2020; Liu et al., 2022; Ma et al., 2019; Poi et al., 2022; Schockett et al., 2021; Shemme et al., 2022; Vig, 2019  Braus et al., 2016; Hernandez-Zambrano et al., 2024 |
| SPC Operational | - Early/automatic referral if positive screened - Structured protocols - Algorithms or pathways - Hospice-supported care - Hospice-supported or step-down palliative pathway - Routine palliative care consultation within 48 hours of ICU admission - Proactive case-finding by Specialist ANPs - Screening resulted in ICU physician consideration for SPC consultation | Cox et al., 2018; Grouls et al., 2022; Henderson et al., 2017; Ma et al., 2019; Morris et al., 2021; Shemme et al., 2022; Metaxa et al., 2021; O'Mahony et al., 2017; Zalenski et al., 2017 |
| Palliative-Led/Support Family Meetings | - Scheduled family meetings - Shared decision-making - SPC Joint meetings - Leaflets aimed to support families during the post-ICU bereavement period - Identification of family emotional needs | Carson et al., 2016; Ma et al., 2019; Martz et al., 2020; Poi et al., 2022; Shemme et al., 2022; Kim et al., 2022 |
| SPC & Symptom Assessment & Management | - Advanced symptom assessment & management by SPC - SPC management of refractory symptoms - SPC established terminal extubating and terminal discharge protocols to facilitate care transitions - Social, emotional, and spiritual interventions by SPC - SPC or ANP Identification of emotional needs - GOC discussions & Care plan created by SPC - SPC involved in early GOC conversations - Consults available for complex cases | Chung et al., 2022; Henderson et al., 2017; Poi et al., 2022; Shemme et al., 2022; Ma et al., 2019;  Mun et al., 2016; Dolmans et al., 2023 |
| **SPC Operational Processes** | - **Structured Collaborations**    - Neurocritical Care (NCC) and Neuropalliative Care (NPC) collaborative model was introduced   - NCC specialist was stationed in the ICU, providing close monitoring and interaction with patients, families, and care team   - Collaborative phone or web-based conference once referral made   - Joint decision making   - Weekly meetings   - Multidisciplinary working group   - Consults available for complex cases   - Recommendations and coaching to promote primary skills   - Encouraged meetings   - PCplanner app facilitated collaboration between ICU teams & SPC | Liu et al., 2022; Poi et al., 2022; Shemme et al., 2022 |
|  | - **Workflow routine**    - GOC discussions   - Joint decision making   - Weekly meetings   - Multidisciplinary working group   - Consults available for complex cases   - Team huddles   - Recommendations and coaching to promote primary skills, encouraged meetings;   - PCplanner app facilitated collaboration between ICU teams & SPC   - The program was integrated into the existing inpatient palliative care consultation service | Chung et al., 2022; Cralley et al. 2022; Cox et al., 2018: Davila et al., 2023; Frontera et al., 2015; Grouls et al., 2022; Liu et al., 2022; Ma et al., 2019; Paré et al., 2021; Poi et al., 2022; Schockett et al., 2022 |
|  | - **Operational process**   - Early consultation   - Structured referral processes   - Feedback loops   - COVID ECMO pathway   - Role playing   - Champions   - Checklist screening | Grouls et al., 2022; Cox et al., 2018; Metaxa et al., 2021 |
|  | - **Role Clarity and Collaborative Balance:**    - Excessive dependence on specialist may lead to fragmented care and reduce the motivation | Anderson et al., 2017; Aslakson et al., 2017; Gupta et al., 2022; Mehta et al., 2023; Mun et al., 2018 |
| **Recommendations for SPC Collaboration** | - Expand SPC roles - Tailored communication - Hybrid models | Anderson et al., 2017; Gupta et al., 2022 |
| **MDT Across Specialties** | - Chaplains, psychologists, social workers, physicians; enhanced communication, multidisciplinary working group | Chung et al., 2022; Cralley et al. 2022; Shemme et al., 2022;  Braus et al., 2016; Poi et al., 2022 |
| **Care continuity** | - Care continuity post ICU by SPC | Poi et al., 2022; Shemme et al., 2022 |
